# An Interactive Workshop to Enhance Teaching Skills Through Understanding Teaching Styles

**DOI:** 10.15766/mep_2374-8265.11571

**Published:** 2026-01-20

**Authors:** Adin Nelson, Suzanne Friedman, Elizabeth Goodman, TJ Jirasevijinda, Meghan Treitz, Helen Wang, Adam Weinstein

**Affiliations:** 1 Associate Professor, Department of Pediatrics, Weill Cornell Medicine; 2 Associate Professor, Department of Pediatrics, Columbia University Vagelos College of Physicians and Surgeons; 3 Associate Professor, Department of Pediatrics, Rutgers Robert Wood Johnson Medical School; 4 Professor, Department of Pediatrics, Weill Cornell Medicine; 5 Associate Professor, Department of Pediatrics, University of Colorado Anschutz Medical Campus; 6 Associate Professor, Department of Pediatrics, Stanford University School of Medicine; 7 Professor, Department of Medical Sciences and Pediatrics, Frank H. Netter MD School of Medicine at Quinnipiac University

**Keywords:** Teaching Styles, Teaching Skills, Educator Development, Faculty Development

## Abstract

**Introduction:**

Teaching is an essential physician competency, yet education on how to teach is limited in medical training and often emphasizes specific skills over basic approaches. We developed a workshop to help educators understand their inherent teaching styles and explore strategies from other styles using the framework developed by Grasha and Riechmann.

**Methods:**

To maximize participant engagement, we used clips from the Harry Potter films where characters exemplify the different teaching styles. We described the strengths and weaknesses of each style and reviewed the importance of matching teaching style to context and learners’ needs. Participants then discussed various teaching scenarios in small groups to deepen their understanding of their default natural teaching style and to explore less-familiar teaching styles. We evaluated the workshop using surveys with Likert scales and narrative feedback. All authors collaborated on inductive thematic analysis of written comments.

**Results:**

We delivered this workshop at seven regional, national, and international conferences. Of 170 respondents, 99% reported understanding their natural teaching style better, and 91% indicated they would likely change their teaching approach after participating. We identified three themes in participants’ feedback: making conscious choices about teaching styles, the value of a flexible approach that incorporates elements from the full range of teaching styles, and the importance of matching teaching style to learners’ specific needs and the educational context.

**Discussion:**

This workshop complements existing materials on specific teaching skills by providing a foundational understanding of teaching styles. Using excerpts from popular movies makes the workshop both entertaining and memorable.

## Educational Objectives

By the end of this activity, learners will be able to:

1. Identify and understand their natural teaching style using the framework described by Grasha and Riechmann.
2. Compare and contrast the five teaching styles described by Grasha and Riechmann.
3. Incorporate elements from all five teaching styles intentionally and flexibly to target particular learner needs and situations.

## Introduction

All physicians teach in some capacity, whether it is to educate patients or non-physician colleagues, or as academic physicians with a formal role in education. Undergraduate and graduate medical education programs typically prioritize clinical and research training over teaching; however, in recent years, there have been increasing calls for medical training programs to devote more time to teaching skills.^1,2^ Residency training programs are now required to provide education about teaching,^3^ but resident-as-teacher curricula and faculty development programs tend to focus on building concrete skills such as small-group facilitation, procedure teaching, or feedback and evaluation.^4–6^ Less attention has been paid to educators’ own teaching style and the ways in which it might impact how they teach and interact with learners.

Teaching styles represent the inherent, preferred ways that people teach. Without a thorough understanding of teaching styles, their own natural style, and the benefits and disadvantages of each, trainees and junior faculty may be left to teach based on imitating their role models and discovering what comes to them naturally.^7,8^ Several published frameworks describe different teaching styles,^8–10^ all of which are underpinned and supported by educational philosophies that represent the beliefs and values educators ascribe.^11,12^ By understanding their own teaching style and learning about others, educators can work more effectively with learners, build stronger relationships, and develop their own skills.

We chose the framework described by Anthony Grasha and Sheryl Riechmann^10^ for its broad applicability across the diverse contexts in which physicians teach. Grasha and Riechmann observed college professors teaching and developed a framework of five different teaching styles that described the behaviors they observed. Their work was entirely observational and explicitly excluded analysis of the relative effectiveness of the different styles. The five Grasha-Riechmann teaching styles are as follows: Expert, Formal Authority, Personal Model, Facilitator, and Delegator. Expert teaching is grounded in specific knowledge and/or skills that one person—the teacher—has and another person—the learner—needs; the teacher shares their expertise, and the learner is expected to absorb it. Formal authority teaching is grounded in the position of power that the teacher holds over the learner(s); the teacher uses that position to set clear expectations and create an organized learning structure. Personal model teaching involves teaching by example; the teacher demonstrates while the learners observe, then the teacher observes and provides feedback while the learners practice. Facilitator teaching relies on strong teacher–learner relationships, allowing the teacher to guide learners through their own individual learning. Facilitator teachers function more as coaches than as instructors. Delegator teaching emphasizes learners’ autonomy by assigning learning tasks and then leaving them to work independently. A key tenet of Grasha and Riechmann's framework is that all five styles are useful, effective, and appropriate; no one style is better than the others, despite any pressures of social desirability in different disciplines or at different times. More detailed descriptions, examples, and analyses of the strengths and weaknesses of each style can be found in Appendix A.

Teaching styles and educational philosophies are inherently abstract, which can make them less engaging to busy frontline clinical teachers and challenging to apply in everyday use. We developed a workshop to help clinician-educators understand their natural teaching style and learn about other teaching styles to broaden their own styles for different clinical and educational scenarios. Our goal was to help workshop participants recognize their instinctive teaching style, identify strengths and weaknesses of their usual default teaching style, and enhance their approach to teaching by thoughtfully incorporating elements of teaching styles that do not align with their natural tendencies. An additional aim was to emphasize that while these styles are grounded in educational philosophy and evidence, none is inherently superior to any other, and to help participants see that teaching can be most effective when they can adapt their style to their particular learners and setting.^7^

We designed our workshop to be relevant for clinician-educators at all career stages, from trainees and junior faculty who are developing their teaching style to senior educators with established teaching styles who would benefit from an opportunity to reflect on and share what they do. To our knowledge, this is the first published workshop in medical education focused on underlying teaching styles rather than higher-level concrete teaching skills. A more nuanced understanding of teaching styles can support educators throughout their careers, providing a solid foundation upon which they can build their skills, as opposed to learning individual skills only appropriate for specific contexts. As educators may revert to their default teaching styles when they are busy, stressed, or in new situations, it is therefore imperative for clinician-educators to understand those styles for maximal success.^8^ To bolster workshop participants’ engagement and learning, we used well-known fictional teachers as examples for each of the teaching styles. Selected scenes from the Harry Potter movies were used to illustrate many portrayals of different teaching styles, leveraging the films’ popularity to enhance engagement and to allow for diverse representations of styles, given the teaching-heavy setting of the plot. Based on the theory of multimedia learning, seeing the teaching styles portrayed in movie clips—rather than just reading descriptions of them—boosts learning,^13,14^ and associating each teaching style with a well-known fictional character aids memory and adds an element of humor to the workshop.

## Methods

We delivered this workshop at multiple regional, national, and international conferences including the Council on Medical Student Education in Pediatrics (2023), the Pediatric Academic Societies (2023), the Association of Pediatric Program Directors (2024), the Association of American Medical Colleges Northeast Group on Educational Affairs (2024), the Stanford Medical Education Forum (2024), the International Conference on Residency Education (2024), and the International Conference on Residency Education Program Administrators’ Pre-Conference (2024). All facilitators studied the Grasha-Riechmann framework of teaching styles in advance. We identified a specific character from the Harry Potter movies who exemplified each teaching style and chose a movie clip that demonstrated that style. We did not assign workshop participants preparatory work, and we set the explicit expectation that no prior knowledge of the Harry Potter series was required. While we anticipated that most attendees would already be familiar with the Harry Potter movies, we included movie clips and detailed explanations so that the workshop content would be self-contained.

The workshop included both didactic presentations and small-group discussions. After introducing the workshop, setting goals and educational objectives, and reviewing disclosures, we invited participants to share a recent challenging teaching encounter and how they approached it. We then presented an overview of the concept of teaching styles and focused explanations of each of the five teaching styles. For each teaching style, we introduced and defined it, shared strengths and challenges of using that style, identified a Harry Potter movie character who typified it, and showed a video clip of that character demonstrating that teaching style. Both our institutional legal counsel and the organizers of the conferences confirmed that we could show brief excerpts from the movies for this educational, noncommercial, purpose. The slides for the workshop, including instructions on where to find the movie clips, are in Appendix B.

After introducing all five teaching styles, we asked participants to identify which teaching style felt most natural to them and instructed them to form groups with others who shared the same natural teaching style. We then distributed brief written summaries of challenging teaching scenarios (Appendix C) and asked participants to discuss in their groups how they would approach those scenarios. For the first set of breakout groups, we instructed participants to focus on how they would manage the teaching scenarios through their identified default teaching style. After allowing time for group discussion, we invited a volunteer from each group to share their chosen teaching style and explain how they would approach each scenario in that style. Of interest, the exercise also engendered discussion of how some people's default teaching style was not necessarily well-suited to a particular scenario. During the small-group discussions, the facilitators circulated throughout the room to provide support and encouragement for participants. The authors are all pediatricians, so the cases we used (Appendix C) all reflect that context. Users are encouraged to adapt these materials by substituting teaching scenarios that better reflect their own clinical context. Appendix D (the facilitator guide) provides suggestions.

Following the initial breakout groups, we delivered a brief didactic presentation on the importance of adaptability for effective teaching and the necessity of assessing learners’ needs and tailoring teaching styles to the scenario and environment. We then reorganized the breakout groups to create new combinations of participants, with as many natural teaching styles as possible represented in each group. We aimed to have at least one representative of each teaching style in each group; however, that proved challenging with some audiences, as participants identified with some styles far more commonly than others. The second set of teaching scenarios (Appendix C) was distributed, and participants were asked to discuss how they would approach each scenario. For these mixed teaching styles breakout groups, we also instructed participants to first share their own natural teaching style and how they would approach the scenario in that style, followed by a discussion of which elements from other style(s) might be more effective for that scenario. Facilitators circulated throughout the room to support group discussions as needed. After the small-group discussion, we asked volunteers to share how their group chose to manage each scenario, and we facilitated a broader conversation about the relative merits of the different teaching styles in each situation. Appendix D (the facilitator guide) includes sample responses of how someone using each style might approach each scenario.

Finally, we wrapped up the workshop by inviting participants to revisit a recent challenging teaching scenario—either the one they had identified earlier or a different one—and reflect on how their approach might change moving forward, informed by the insights and strategies explored during the workshop. We then prompted all participants to complete the workshop evaluation (Appendix E) through an anonymous online survey form. At the end of the workshop, we distributed a resource packet (Appendix A) with information about the teaching styles and key references.

The workshop evaluation included both quantitative measures of participants’ perceptions and open-ended narrative questions to explore what participants learned during the workshop and how they might change their practices because of the workshop. For the quantitative questions, we chose a retrospective pre/post workshop framework rather than separate pre- and postworkshop surveys to save time and to avoid the pitfalls of response-shift bias, and framing and recall bias inherent in using the same questions before and after the session.^15^ We piloted these questions and answer choices and made revisions to ensure that all participants would interpret them as intended. We distributed the evaluations as a digital Qualtrics survey by sharing a QR code at the end of the workshop and allotted 5 minutes for participants to complete the survey before leaving the room. For the Likert-style multiple-choice questions, we chose 4-point rating scales in which respondents had to select their preferred answer. We examined the data quantitatively with descriptive statistics. We then conducted a thematic analysis of narrative comments using a manual approach in a shared Google document. All authors read through participant comments, highlighting meaningful phrases and annotating them with comment boxes to assign initial codes. We identified themes by consensus.^16^

When we presented this workshop at regional, national, and international conferences, the duration of the workshop was between 75 and 90 minutes long. Especially for a larger group of participants, we believe this workshop could easily be extended to 120 minutes by allowing more time for discussion and debriefing with the small groups. We have also presented a shortened 60-minute version of this workshop many times in local institutional settings. Appendix D includes 60-, 75-, and 90-minute versions of the timeline for the workshop. Subjectively, the workshop was well-received in its shortened form, but we did not include the evaluation data from those sessions in our analysis as there were too few responses.

The Weill Cornell Medicine Institutional Review Board (IRB) determined that this project consisted of analyzing only anonymous data generated through standard educational practices, and that it therefore did not meet the regulatory definition of human subjects research and did not require further review or approval (IRB No. 25-05028840-01).

## Results

We delivered this workshop at multiple regional, national, and international conferences and in local institutional settings. The workshops were well attended, with a total attendance of more than 200 participants. We did not count the number of individuals who attended each session, so we are unable to report the total number of learners involved per session. We also did not collect demographic data about the participants, and we did not record participants’ specialties, level of training, or natural teaching style.

Overall, 170 individuals completed the workshop evaluation. 99% of respondents reported that they understood their own natural teaching style better after the workshop than before the workshop, with 55% selecting *much better than before* and 44% selecting *slightly better than before*. A total of 91% of respondents reported that they were likely to change how they taught after participating in the workshop, with 31% responding *definitely yes* and 60% responding *probably yes*.

Twenty individuals provided narrative feedback on the workshop evaluation. Our qualitative review of participants’ narrative responses revealed three central themes reflecting their perceptions of the workshop: (1) intentionality about teaching styles, (2) flexibility about teaching styles, and (3) targeting teaching styles to learners and situations.

### Intentionality About Teaching Styles

Workshop participants noted that they gained insight into the value of making conscious and intentional choices about their teaching style rather than relying on a fixed approach without regard for the context. Sample quotations include: “I will think more intentionally about the style of teaching I am using and why” and “[I will] be mindful of the different teaching styles and not always default to the 1–2 in which I'm most comfortable.” Participants specifically shared that learning about the different teaching styles described by Grasha and Riechmann gave them the tools and the mental vocabulary to be able to make more deliberate choices. For example, one participant stated, “I'll be more aware of my style and other styles that could work.” Another participant commented, “The Grasha & Riechmann framework is VERY helpful nomenclature!”

### Flexibility About Teaching Styles

Another major theme in the participants’ written feedback was that it is not only acceptable but also essential to be flexible about how one teaches and to incorporate elements of many different teaching styles. After the workshop, participants reported a reinforced belief that all the different teaching styles have merit and are equally valid. Sample comments include: “I'm more comfortable with being flexible between teaching styles based on situation and learner. Before, I saw this as more disingenuous or inauthentic, but now I understand that it is part of effective teaching”; and “I think I will just be more comfortable changing styles, and not worry if I use an expert style, even if it doesn't match my teaching philosophy.”

Many participants also specifically commented that this workshop helped them understand that all teaching styles are effective and appropriate, even though some may superficially seem to be at odds with the culture of medical education. For example, one participant commented, “I tend to think of formal authority teaching as more negative in Med Ed, but I understand now it has its place.” Another participant stated, “There is a place for the expert [teaching style]—I initially thought I had to be that, but never felt I was going to get there—then I learned expert [teaching] may not be appropriate in modern Med Ed. Now I see how it all fits together.”

### Targeting Teaching Styles to Learners and Situations

Participants additionally commented on the importance of targeting or tailoring one's teaching style to the specific learners, subject, and context. Beyond acknowledging in principle that all five teaching styles are useful, participants learned that they could enhance their own teaching by intentionally and explicitly shifting into different styles in different situations. Example comments include: “I will adapt my teaching style to each learner and each situation rather than being rigid about how I teach and trying to teach the same way for everyone”; and “[I will] more clearly match a situation with the appropriate teaching style or combination of styles.”

In addition to the comments that reflected on our educational objectives, participants also provided feedback on the timing, logistics, and didactic explanations in the workshop. The version presented herein is the final version we arrived at after incorporating participants’ feedback, reflecting a series of iterative refinements made across multiple presentations of the workshop. Participants also commented favorably on how much they enjoyed the use of the Harry Potter movie clips.

## Discussion

Teaching is an essential skill for all physicians, yet training about teaching often takes a backseat to clinical training and research training in medical education programs.^17–19^ The existing resources on teaching how to teach tend to focus on concrete skills rather than the underlying principles of how to approach teaching. For example, within *MedEdPORTAL*’s published collection on teaching skills, both for faculty development and for resident-as-teacher programs, the focus is on training how to teach many of the ACGME competencies and procedural skills. None of them explicitly addresses self-reflection on approaches to or styles of teaching. We believe this workshop adds to medical education literature, specifically *MedEdPORTAL* resources, by sharing a robust framework and familiar examples that can help medical teachers identify, understand, and adjust their natural teaching style, in a concrete, engaging, and entertaining way. We have delivered this workshop to groups of mixed health professionals, physicians from many specialties, and individuals with widely varied levels of teaching experience, and the feedback has been uniformly positive from all groups.

We believe that using examples based on the characters and clips from the Harry Potter movies is a key contributor to the effectiveness of this workshop. First, the workshop is entertaining. Including dynamic clips from popular media enhances participation. Second, interspersing mini-lectures, video clips, and hands-on activities boosts participants’ attention and focus by thoughtfully changing the format every few minutes. Third, the workshop aids retention. For participants familiar with the Harry Potter series, the materials and activities are likely more memorable to them. Even for participants who did not know the Harry Potter series, multimedia learning theory^13^ suggests that the video clips coupled with hands-on activities should be effective memory aids.

We acknowledge that relying heavily on the fictional characters from the Harry Potter movies as examples may be a limitation. We included video excerpts from the movies along with detailed explanations of the teaching styles, rather than relying on character references, to try to make the workshop self-contained and accessible for those with no prior knowledge of the films or books. In the workshops already presented, which we delivered to diverse participants, we observed that many participants were Harry Potter fans, so we cannot fairly judge how effective the workshop would be for participants without that background. This could have potentially skewed our results due to selection bias. However, the clips we have selected closely mirror the descriptions of the different teaching styles in Grasha and Riechmann's model, such that it was easy to understand and apply for those who were not Harry Potter fans. Conceivably, it could take more preparation for an educator who does not already know the characters to prepare this workshop. Lastly, our results are subject to recall bias as participants submitted their feedback at various time intervals after their workshop.

We implemented this workshop with diverse participants, including learners of different levels and from different disciplines, and we expected that different natural teaching styles might predominate in different groups, including, for example, Formal Authority Teaching in Surgery, Expert Teaching in Internal Medicine, or Facilitator Teaching in Pediatrics. While we did not record participants’ demographic information or natural teaching styles, we found that our expectations were unfounded; our subjective general impression was that all teaching styles were represented in all groups. Similarly, as facilitators, we learned that we needed to remain open-minded about the different teaching styles and not assume that the most learner-centered one is always the most helpful. For example, Formal Authority may work best when the director of a clinical rotation orients her students at the beginning of the course. One possible future step for this workshop would be to replace the Harry Potter context with a different set of well-known fictional educators to appeal to a different demographic of participants.

Despite the limitations and context-specificity of this workshop, our evaluation data suggest that this workshop is highly effective for participants in a variety of fields of medicine. We are confident that the material would translate equally well to broader health care and educational settings.

## Appendices


Harry Potter Teaching Styles Handout.docxHarry Potter Teaching Styles Workshop.pptxDiscussion Cases.docxFacilitator Guide.docxWorkshop Evaluation.docx
*All appendices are peer reviewed as integral parts of the Original Publication.*

## References

[1] Dandavino M, Snell L, Wiseman J. Why medical students should learn how to teach. Med Teach. 2007;29(6):558–565. 10.1080/01421590701477449 17922358

[2] Soriano RP, Blatt B, Coplit L, et al. Teaching medical students how to teach: a national survey of students-as-teachers programs in U.S. medical schools. Acad Med. 2010;85(11):1725–1731. 10.1097/ACM.0b013e3181f53273 20881824

[3] Accreditation Council for Graduate Medical Education. ACGME Common Program Requirements (Residency). ACGME. Accessed December 22, 2025. https://www.acgme.org/globalassets/pfassets/programrequirements/2025-reformatted-requirements/cprresidency_2025_reformatted.pdf

[4] Fromme HB, Whicker SA, Paik S, et al. Pediatric resident-as-teacher curricula: a national survey of existing programs and future needs. J Grad Med Educ. 2011;3(2):168–175. 10.4300/JGME-D-10-00178.1 22655138 PMC3184906

[5] Kalynych C, Edwards L, West D, Snodgrass C, Zenni E. Tuesday's teaching tips-evaluation and feedback: a spaced education strategy for faculty development. MedEdPORTAL. 2022;18:11281. 10.15766/mep_2374-8265.11281 36475014 PMC9678823

[6] Pourmand K, Nagula S, Keefer L, Shah B. Faculty development workshop for endoscopic teaching techniques. MedEdPORTAL. 2020;16:10960. 10.15766/mep_2374-8265.10960 32995496 PMC7511062

[7] Vaughn LM, Baker RC. Do different pairings of teaching styles and learning styles make a difference? Preceptor and resident perceptions. Teach Learn Med. 2008;20(3):239–247. 10.1080/10401330802199559 18615299

[8] Vaughn L, Baker R. Teaching in the medical setting: balancing teaching styles, learning styles and teaching methods. Med Teach. 2001;23(6):610–612. 10.1080/01421590120091000 12098485

[9] Bibace R, Catlin RJ, Quirk ME, Beattie KA, Slabaugh RC. Teaching styles in the faculty-resident relationship. J Fam Pract. 1981;13(6):895–900. 7310344

[10] Grasha AF. A matter of style: the teacher as expert, formal authority, personal model, facilitator, and delegator. College Teach. 1994;42(4):142–149. 10.1080/87567555.1994.9926845

[11] Kirch SA, Sadofsky MJ. Medical education from a theory-practice-philosophy perspective. Acad Pathol. 2021;8:23742895211010236. 10.1177/23742895211010236 33959676 PMC8060751

[12] Saritas E. Relationship between philosophical preferences of classroom teachers and their teaching styles. Educ Res Rev. 2016;11(16):1533–1541. 10.5897/ERR2016.2787

[13] Mayer RE. Multimedia learning. In: Ross BH, ed. Psychology of Learning and Motivation. Vol 41. Academic Press; 2002:85–139. 10.1016/S0079-7421(02)80005-6

[14] Issa N, Schuller M, Santacaterina S, et al. Applying multimedia design principles enhances learning in medical education. Med Educ. 2011;45(8):818–826. 10.1111/j.1365-2923.2011.03988.x 21752078

[15] Wykowski JH, Starks H. What type of self-assessment is best for your educational activity? A review of pre-post, now-then, and post-only designs. J Gen Intern Med. 2025;40(5):1010–1015. 10.1007/s11606-024-09176-w 39495451 PMC11968623

[16] Braun V, Clarke V. Thematic analysis. In: Maggino F, ed. Encyclopedia of Quality of Life and Well-Being Research. Springer, Cham; 2022:1–7. 10.1007/978-3-319-69909-7_3470-2

[17] Hoffert MM, Passalacqua KD, Haftka-George A, Abreu Lanfranco O, Martin RA. A curriculum for enhancing physician teaching skills: The value of physician-educator partnerships. J Med Educ Curric Dev. 2021;8:23821205211032013 10.1177/23821205211032013 34377837 PMC8323411

[18] Nothman S, Kaffman M, Nave R, Flugelman MY. Survey of faculty development in four Israeli medical schools: clinical faculty development is inadequate and clinical teaching is undervalued in Israeli faculties of medicine. Isr J Health Policy Res. 2021;10(1):10. 10.1186/s13584-021-00438-0 33557931 PMC7871531

[19] da Silva Campos Costa NM. Pedagogical training of medicine professors. Rev Lat Am Enfermagem. 2010;18(1):102–108. 10.1590/S0104-11692010000100016 20428704

