# Supplementary material for: An Interactive Workshop to Enhance Teaching Skills Through Understanding Teaching Styles
Source: MedEdPORTAL. 2026 Jan 20;22:11571. doi: 10.15766/mep_2374-8265.11571 (PMC12816393; doi:10.15766/mep_2374-8265.11571)
Supplement: Supplementary file 1 — Harry Potter Teaching Styles Handout.docxHarry Potter Teaching Styles Workshop.pptxDiscussion Cases.docxFacilitator Guide.docxWorkshop Evaluation.docx [file mep_2374-8265.11571-s001.zip › A. Harry Potter Teaching Styles Handout.docx]

Lessons from the Hogwarts School of Faculty Development

What are our natural teaching styles,

and what can we learn from other teaching styles?

Anthony Grasha and Sheryl Riechman developed a framework describing five distinct teaching styles:^1^


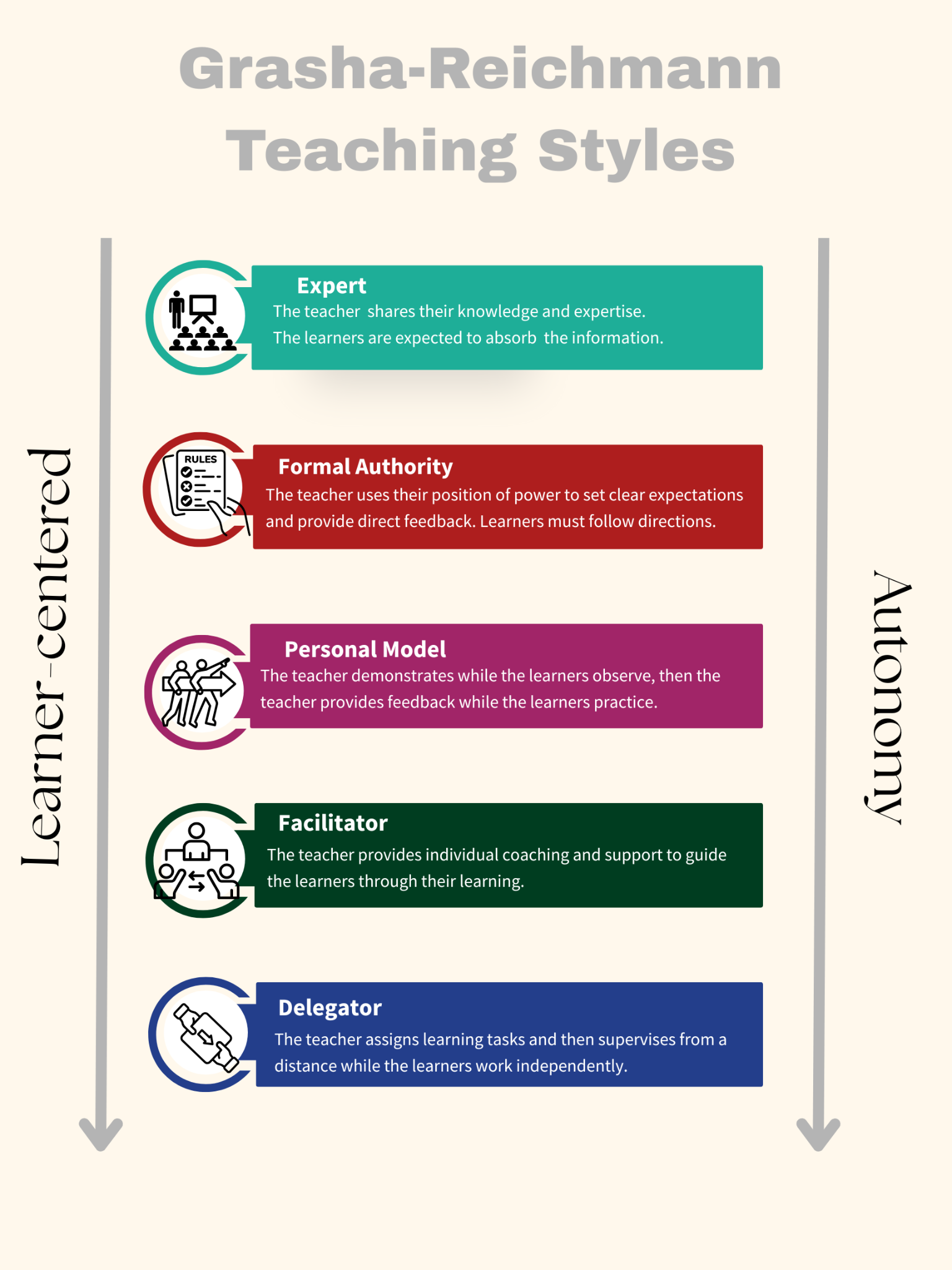


Expert Teaching

Description

The expert teacher possesses knowledge and expertise that students need. Educators who follow the expert style maintain their position as an expert among their learners by demonstrating their detailed knowledge. That challenges the learners to try to build their own skills and expertise to match those of the teacher. The expert teacher concentrates on transmitting information and requires that learners be prepared to learn and use that information.

Advantages

The expert teacher’s wealth of information, knowledge, and skills is the main advantage of this teaching style. This style focuses on transmission of knowledge and skills, and it may also be particularly effective when the educator does not have a position of authority over the learners as in peer teaching.

Disadvantages

If the expert style is overused, the display of knowledge may intimidate learners, especially less-experienced learners. Some educators relying on the expert style may know the correct answers to questions but lack a deeper understanding of the evidence or principles behind them. That can be particularly true for less-experienced educators such as peer and near-peer educators.

Examples

In the Harry Potter movies, Oliver Wood displays expert style teaching when he teaches Harry how to play Quidditch. Another example from the Harry Potter books is Professor Binns who teaches exclusively by lecturing to the students while they take notes. Real-world examples might include peer teaching when one trainee shows another how to accomplish a task or a casual but very knowledgeable attending who teaches simply by sharing their extensive knowledge and skills with residents.

Formal Authority Teaching

Description

The formal authority teacher holds an explicit position of power over their learners, for example: a faculty member to residents or students. Their teaching is grounded in that formal relationship. Formal authority teachers use their position of power to create an organized learning structure by setting clear goals and expectations and providing explicit positive and negative feedback. Learners are expected to follow directions.

Advantages

The formal authority teaching style can set learners up for success by setting clear expectations. This style depends on the educator having an official position of power over their learners.

Disadvantages

Formal authority teaching can be a rigid and inflexible style. Educators who lean heavily on this style may struggle to meet learners’ variable and changing needs. This style can also fail with learners who see themselves as peers or near-peers of the educator.

Examples

In the Harry Potter movies, Professor Snape demonstrates formal authority teaching with his strict classroom management in his first class. Another example from the Harry Potter books (that is not portrayed as clearly in the movies) is Professor McGonagall: she sets very clear expectations about the students’ behavior, and then she gives them explicit feedback on their performance. A common real-life example is a program director or clerkship director going over ground rules during orientation with a new group of learners.

Personal Model Teaching

Description

Personal model teachers teach by role modeling and personal example. This educator provides a prototype for how to think or behave and then oversees, guides, and directs while the learners work to emulate their example. Learners are expected to observe carefully and follow the teacher’s example.

Advantages

A major advantage of the personal model teaching style is that it encourages active learning in which learners practice new skills and “learn by doing,” but it still provides structured guidance through that process.

Disadvantages

Personal model style teaching can fail if the learners are unable to duplicate the skills that the educator demonstrates. Particularly, if the teacher only demonstrates one way of doing

something (the way they do it), they may fail to reach a student who could easily do the task a different way.

Examples

In the Harry Potter movies, Professor Sprout demonstrates personal model teaching when she simultaneously explains and demonstrates what she wants the students to do and then supervises while they practice. Another example is when Hagrid teaches the students about hippogriffs in his Care of Magical Creatures class; the scene demonstrates both how this approach can succeed - when Harry approaches this hippogriff as Hagrid demonstrated - and how this approach can fail - when Draco Malfoy fails to replicate Hagrid’s actions and is attacked. Common real-life examples include trainees learning to do procedures (such as suturing or IV placement) or learning to lead rounds and meetings by watching more seasoned practitioners doing them first.

Facilitator Teaching

Description

Facilitator teaching emphasizes the personal nature of individual teacher-student interactions. The educator guides and directs learners by asking questions, exploring options, and suggesting alternatives as they move together through a collaborative learning process. Educators who use the facilitator style concentrate on the overall goal of developing learners’ capacity for independent action, initiative, and responsibility, while providing support and encouragement.

Advantages

Facilitator style teaching maximizes flexibility by focusing on individual students’ needs and goals. This allows the student to explore options and alternative courses of action. Facilitator teaching also balances students’ independent active learning with ample guidance and support.

Disadvantages

The major challenge of facilitator style teaching is that it requires a strong teacher-student relationship. Developing those personal relationships with every learner can be challenging and time consuming, particularly with large numbers of learners.

Examples

In the Harry Potter movies, Harry himself demonstrates the facilitator style while teaching his peers in Dumbledore’s Army; his teaching is heavily grounded in his individual relationships with the rest of the students. Another example from the Harry Potter books (which is not captured as effectively in the movies) is Professor Lupin working one-on-one with Harry to teach him to cast the Patronus charm; they work closely together, discussing what they are doing and trying different approaches, as Lupin leads Harry through the process of learning. A classic real-life example is an attending coaching a resident or fellow on a complex task: observing, offering feedback, and engaging on an individual level.

Delegator Teaching

Description

Delegator style teaching emphasizes developing learners’ capacity to function independently. Delegator teachers provide basic instructions for a task and then give learners space to explore and learn on their own; the educator steps back from the learning process and serves primarily as a resource on request.

Advantages

The delegator approach to teaching can build learners’ self-confidence by giving them opportunities to be independent. If the learners accomplish their tasks, it sets them up for future success in their own work.

Disadvantages

The major challenge to the delegator style of teaching is that learners are more likely to fail if they are given more autonomy than they are ready to handle. Even learners who ultimately succeed may feel unsupported and overly challenged by this approach.

Examples

In the Harry Potter movies, Professor Slughorn demonstrates delegator style teaching when he assigns the students to produce a particular potion and then steps back to let them try it on their own. Another example from the Harry Potter books (that is not portrayed clearly in the movies) is the way Professor Dumbledore sets Harry up to face extreme challenges on his own and then reappears at the end of the book to debrief Harry’s progress. A common real-life example would be when a teaching attending lets a resident or fellow lead rounds or perform a procedure without direct supervision and then reviews the result with them after the fact.

No one of these teaching styles is more effective than the rest, but some will work better than others in particular situations. Later research by Lisa Vaughn and Raymond Baker showed that matching teaching style and learning style maximizes learning.^2^

^1^Grasha AF. A Matter of Style: The Teacher as Expert, Formal Authority, Personal Model, Facilitator, and Delegator. College Teaching. 1994;42(4):142-149.

^2^Vaughn LM, Baker RC. Do different pairings of teaching styles and learning styles make a difference? Preceptor and resident perceptions. *Teach Learn Med*. 2008;20(3):239-247.
