# Supplementary material for: An Interactive Workshop to Enhance Teaching Skills Through Understanding Teaching Styles
Source: MedEdPORTAL. 2026 Jan 20;22:11571. doi: 10.15766/mep_2374-8265.11571 (PMC12816393; doi:10.15766/mep_2374-8265.11571)

## Slide 1
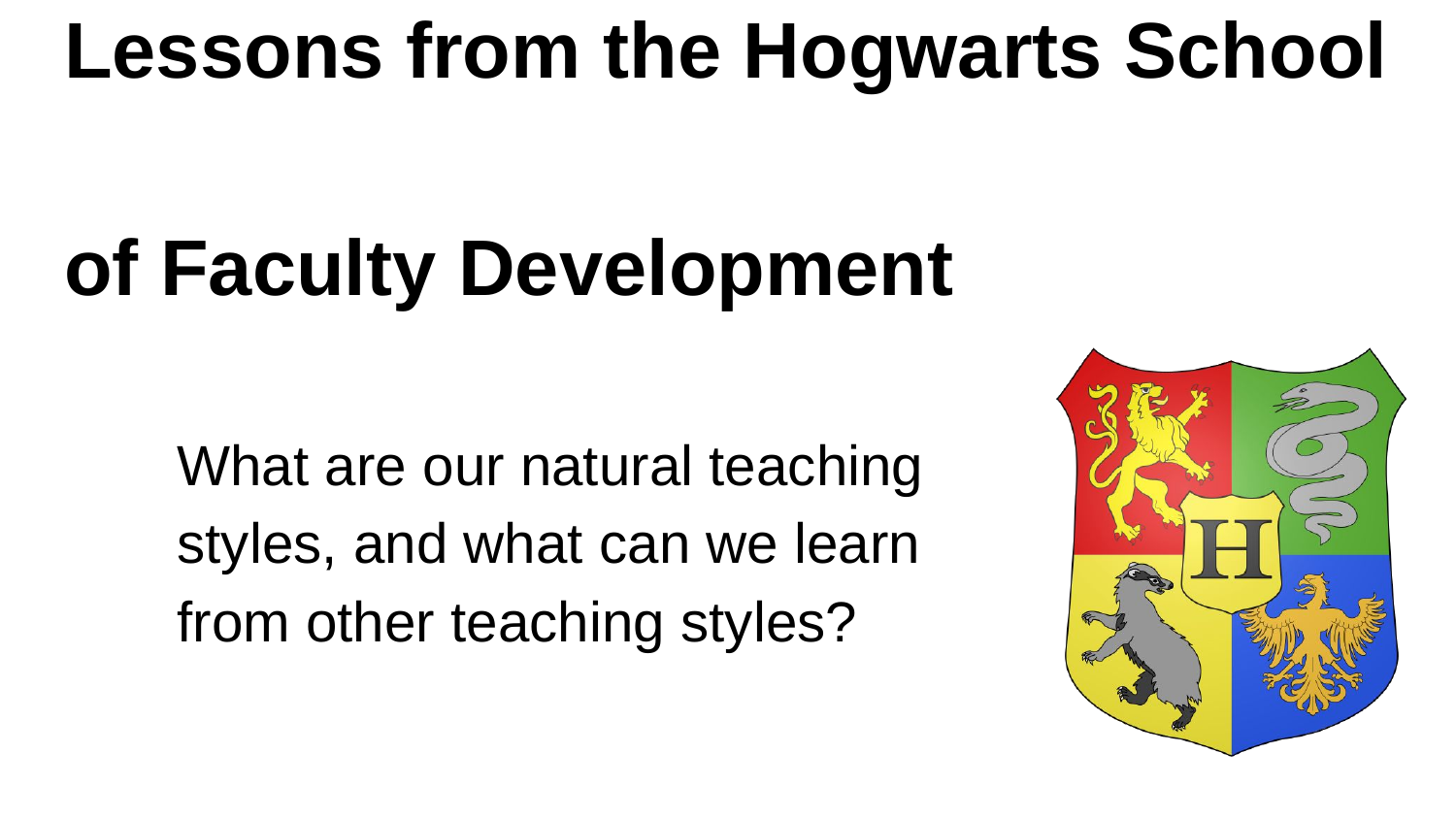

# Lessons from the Hogwarts School of Faculty Development
What are our natural teaching styles, and what can we learn from other teaching styles?

## Slide 2
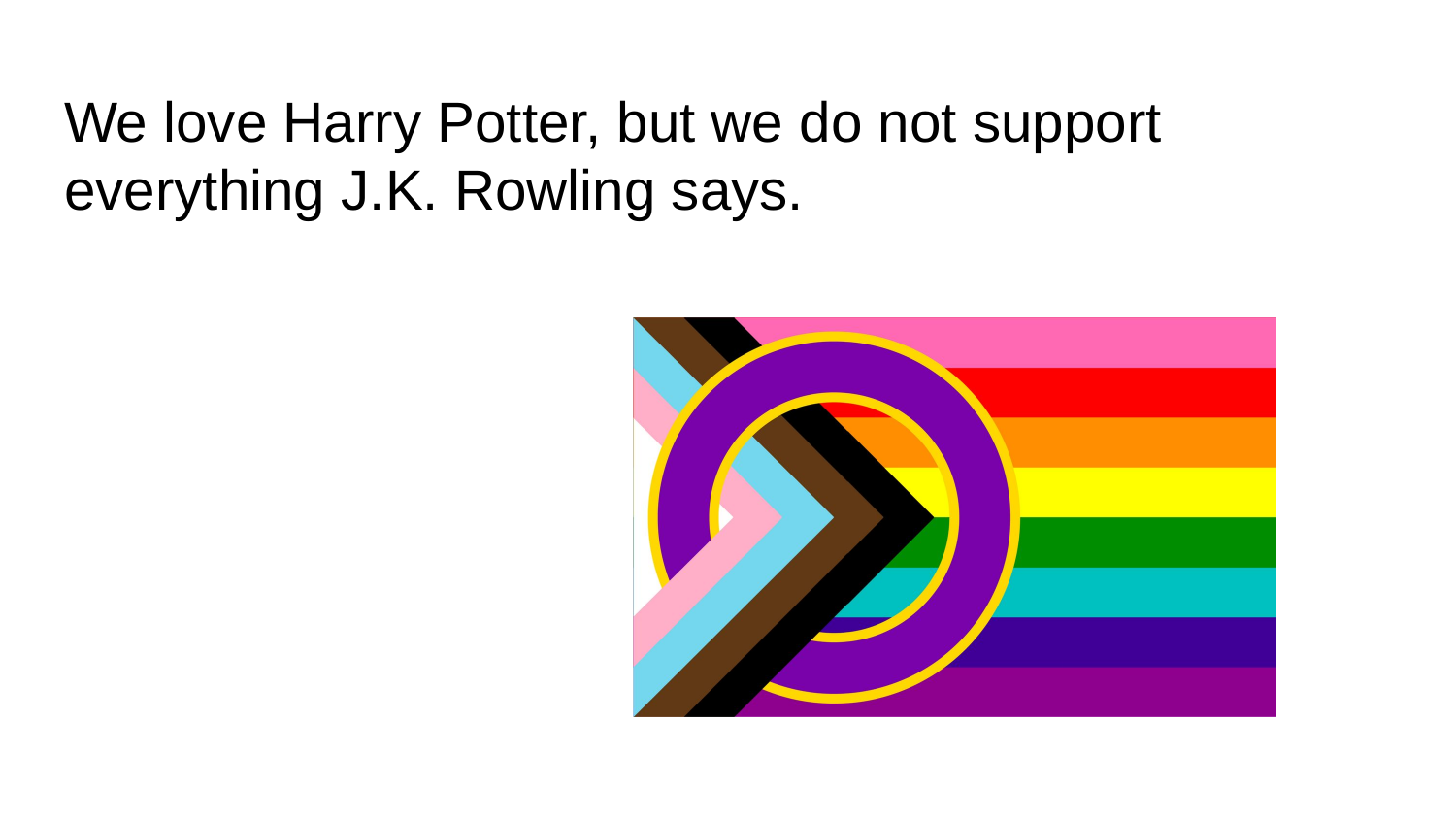

# We love Harry Potter, but we do not support everything J.K. Rowling says.

## Slide 3
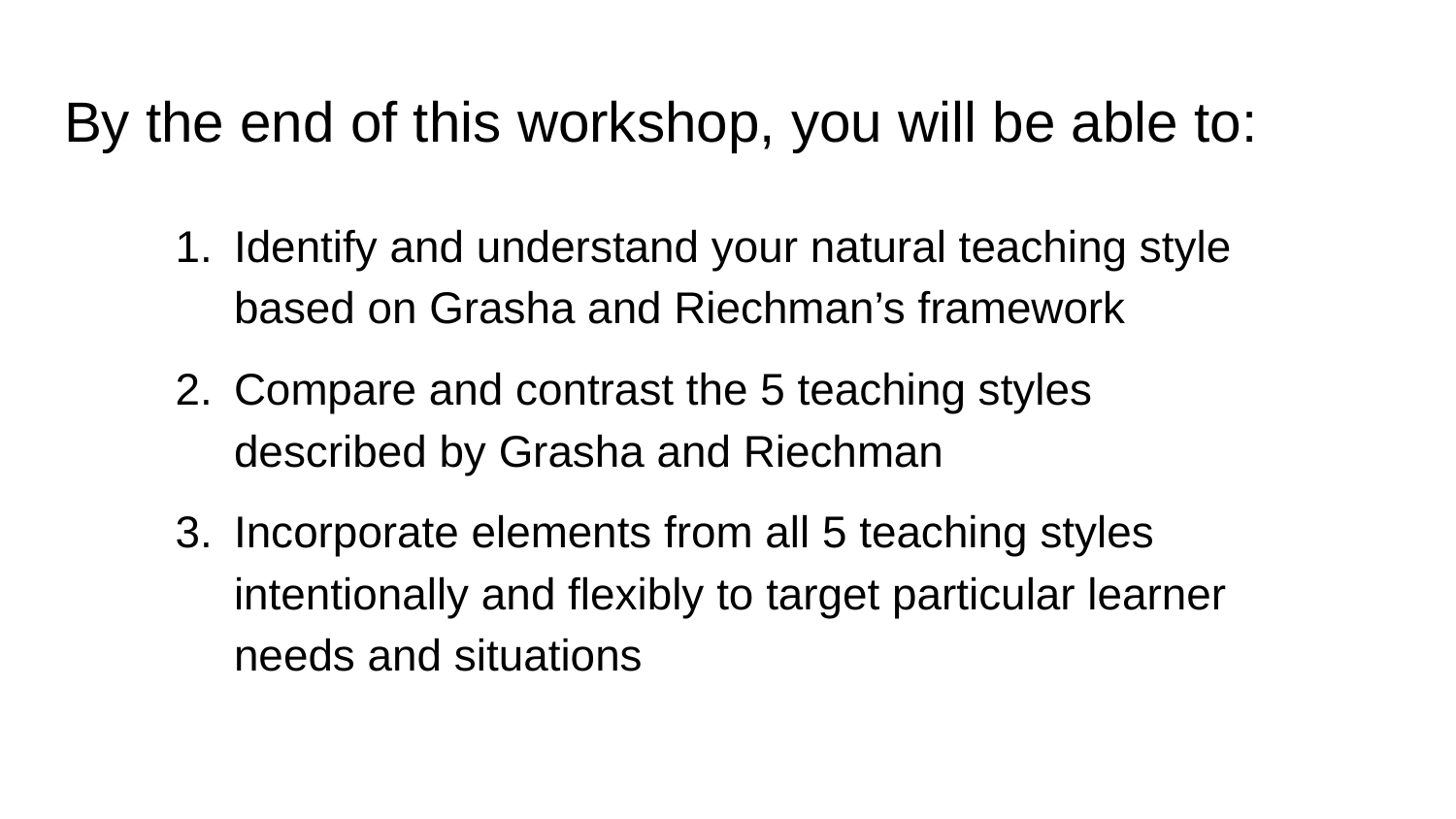

# By the end of this workshop, you will be able to:
Identify and understand your natural teaching style based on Grasha and Riechman’s framework
Compare and contrast the 5 teaching styles described by Grasha and Riechman
Incorporate elements from all 5 teaching styles intentionally and flexibly to target particular learner needs and situations

## Slide 4
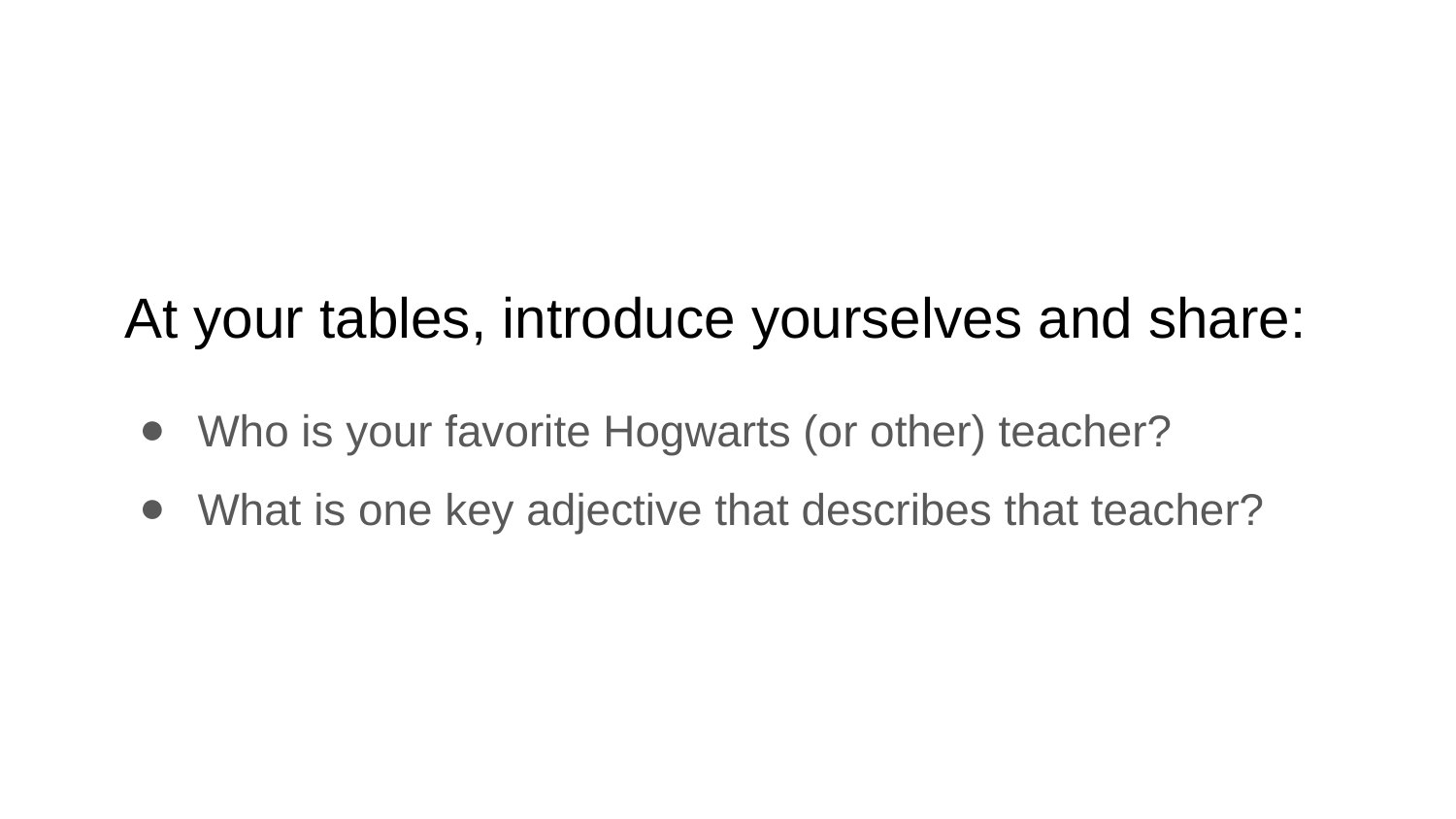

# At your tables, introduce yourselves and share:
Who is your favorite Hogwarts (or other) teacher?
What is one key adjective that describes that teacher?

## Slide 5
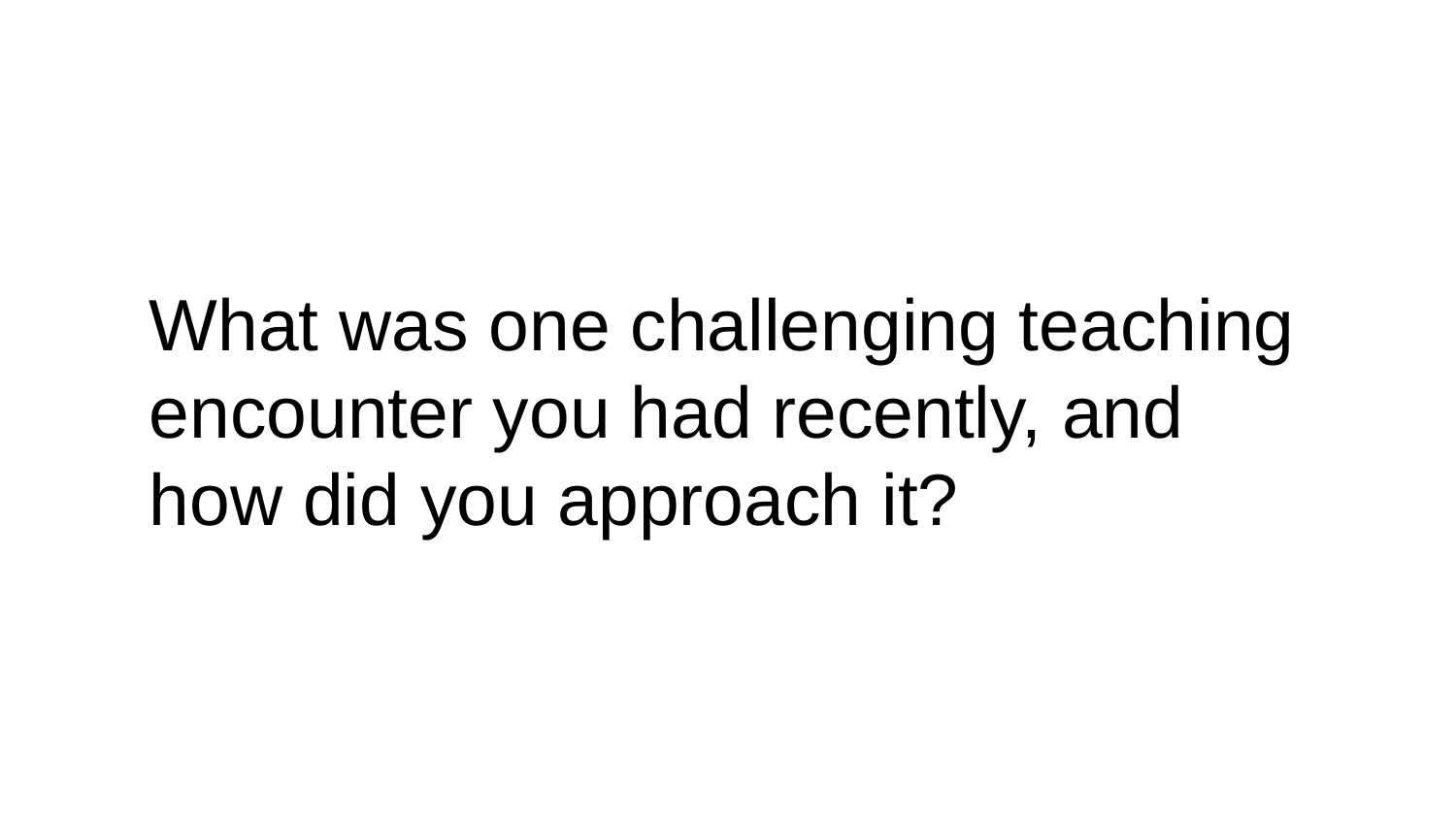

# What was one challenging teaching encounter you had recently, and how did you approach it?

## Slide 6
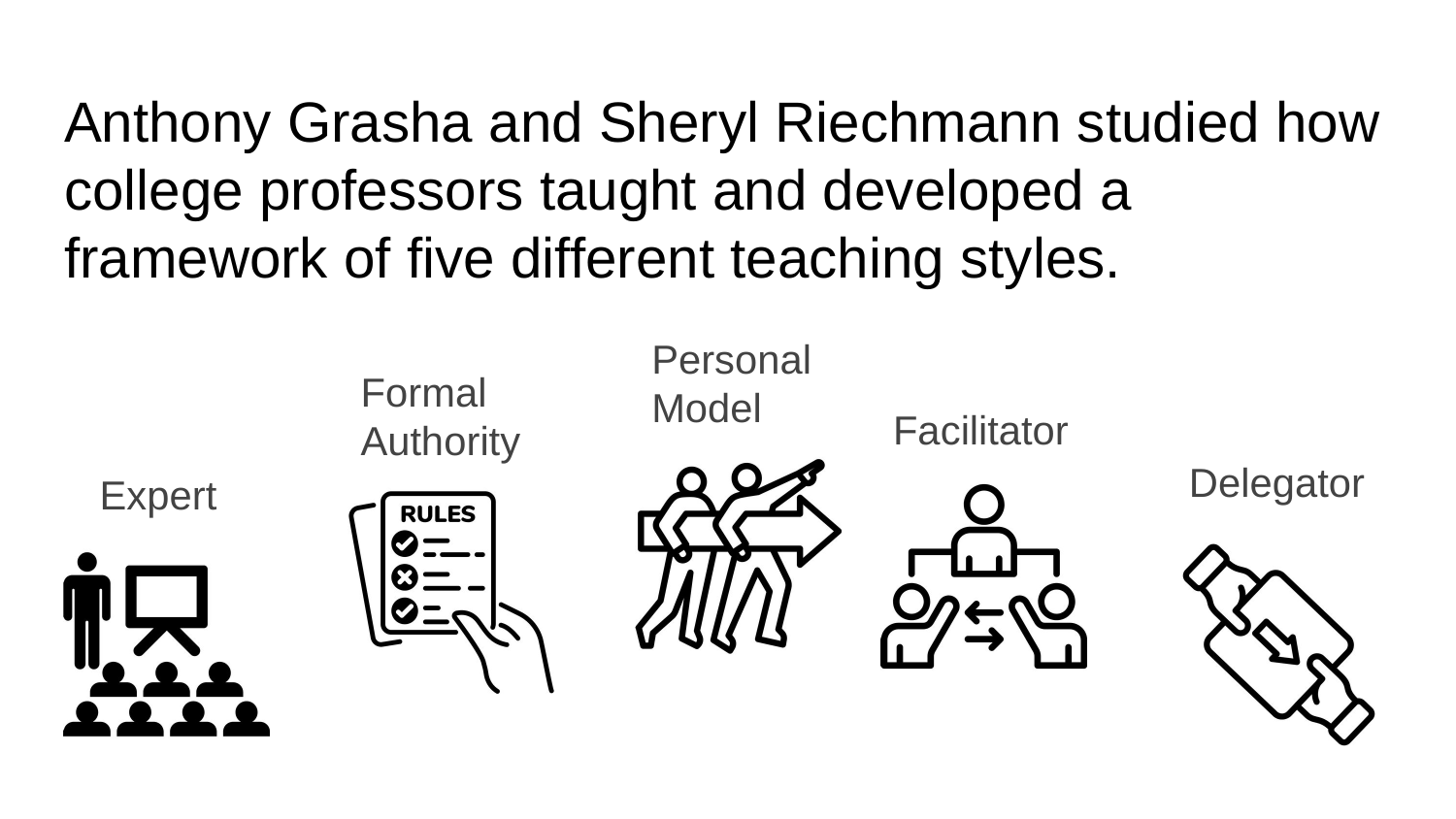

# Anthony Grasha and Sheryl Riechmann studied how college professors taught and developed a framework of five different teaching styles.
Personal Model
Formal Authority
Facilitator
Delegator
Expert

## Slide 7
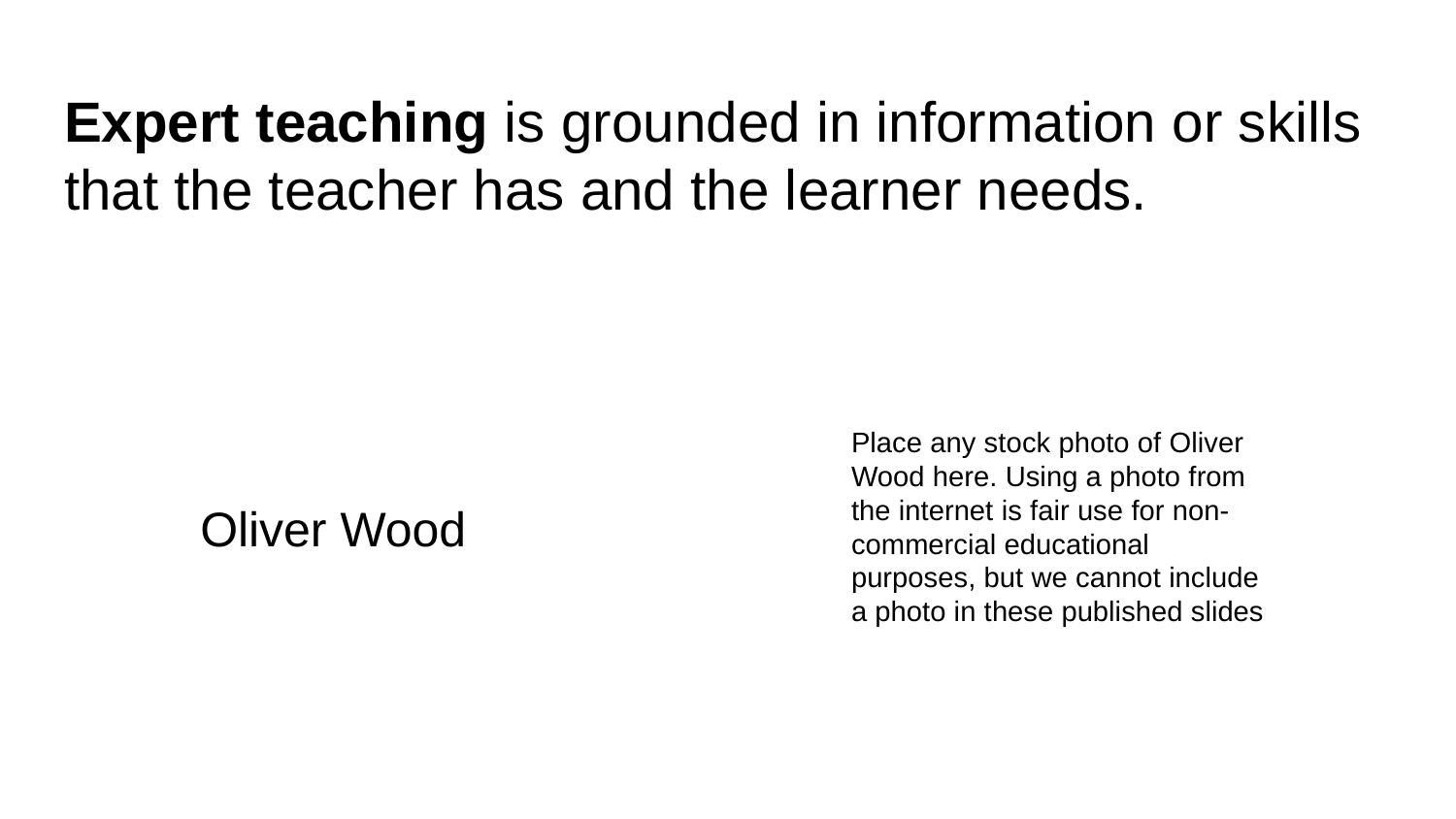

# Expert teaching is grounded in information or skills that the teacher has and the learner needs.
Place any stock photo of Oliver Wood here. Using a photo from the internet is fair use for non-commercial educational purposes, but we cannot include a photo in these published slides
Oliver Wood

## Slide 8
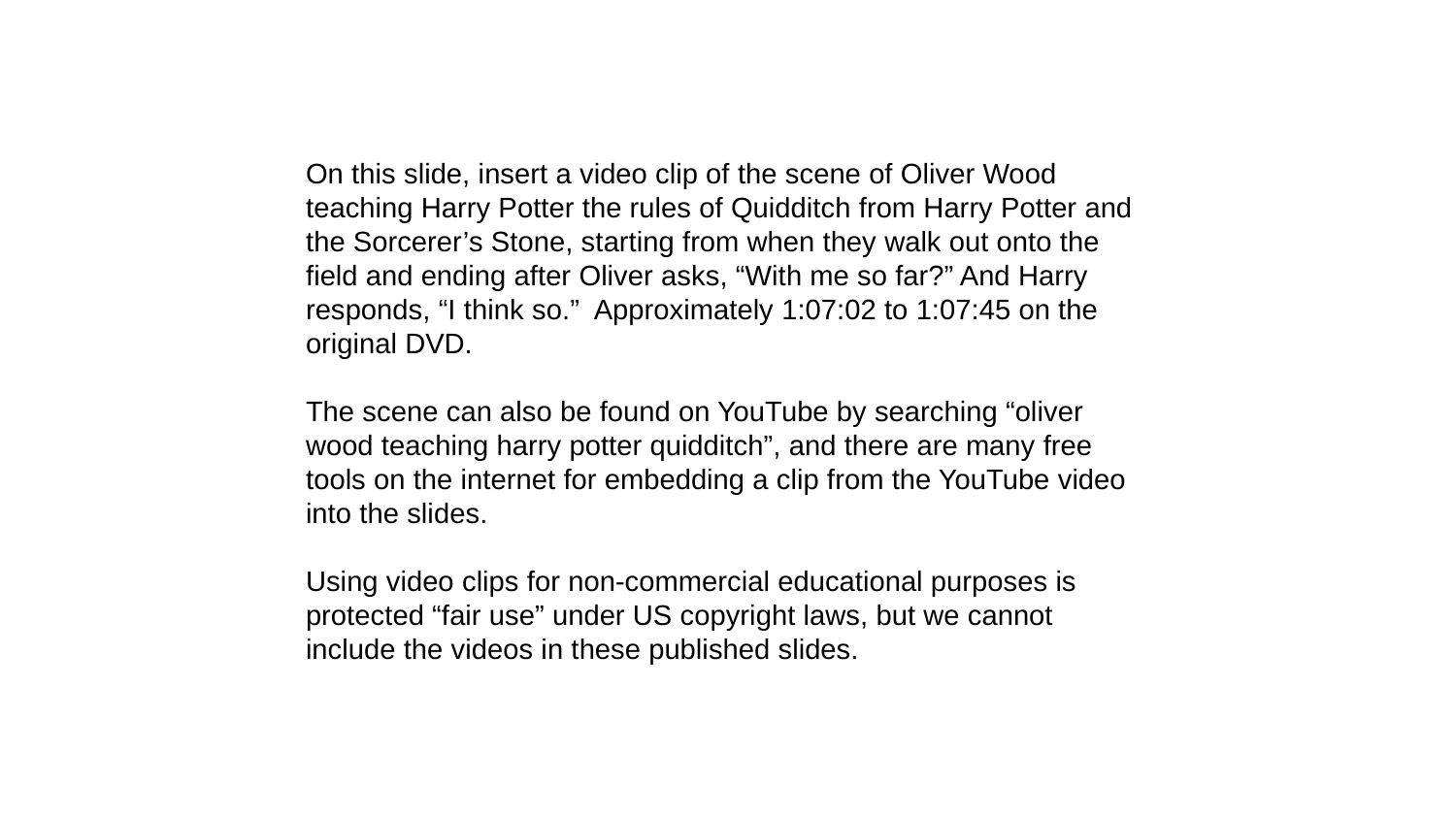

On this slide, insert a video clip of the scene of Oliver Wood teaching Harry Potter the rules of Quidditch from Harry Potter and the Sorcerer’s Stone, starting from when they walk out onto the field and ending after Oliver asks, “With me so far?” And Harry responds, “I think so.” Approximately 1:07:02 to 1:07:45 on the original DVD.
The scene can also be found on YouTube by searching “oliver wood teaching harry potter quidditch”, and there are many free tools on the internet for embedding a clip from the YouTube video into the slides.
Using video clips for non-commercial educational purposes is protected “fair use” under US copyright laws, but we cannot include the videos in these published slides.

## Slide 9
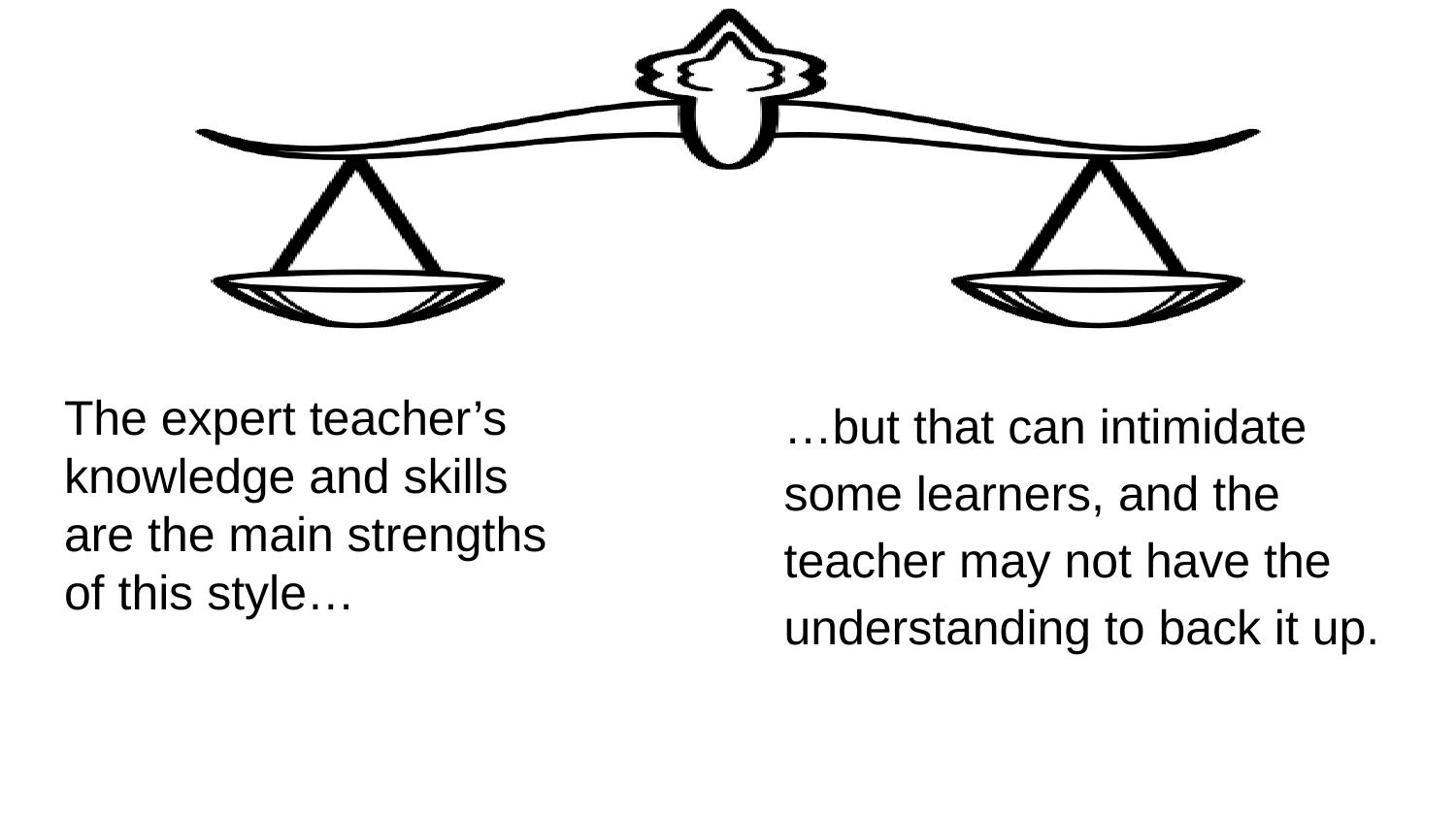

The expert teacher’s knowledge and skills are the main strengths of this style…
…but that can intimidate some learners, and the teacher may not have the understanding to back it up.

## Slide 10
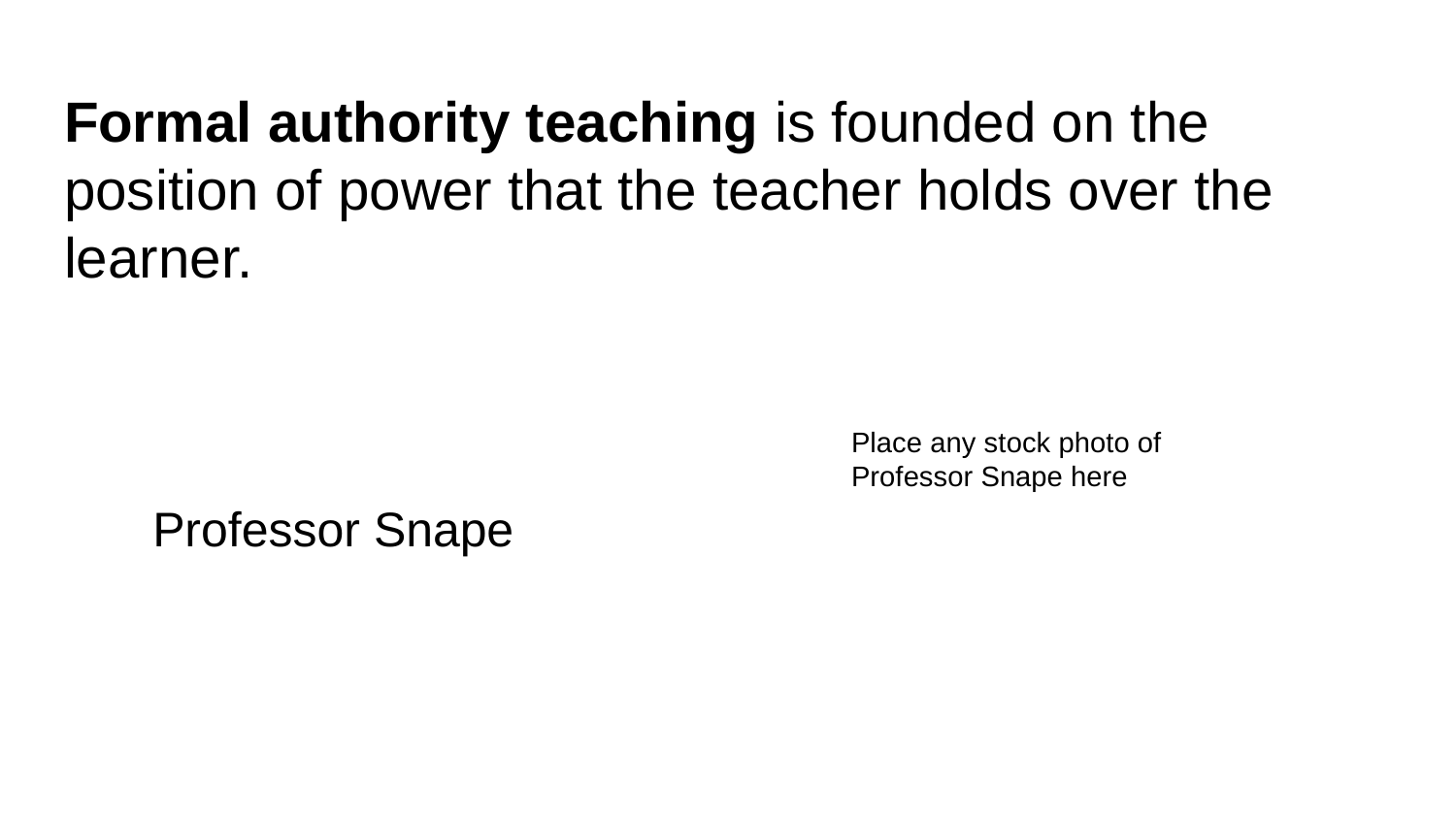

# Formal authority teaching is founded on the position of power that the teacher holds over the learner.
Place any stock photo of Professor Snape here
Professor Snape

## Slide 11
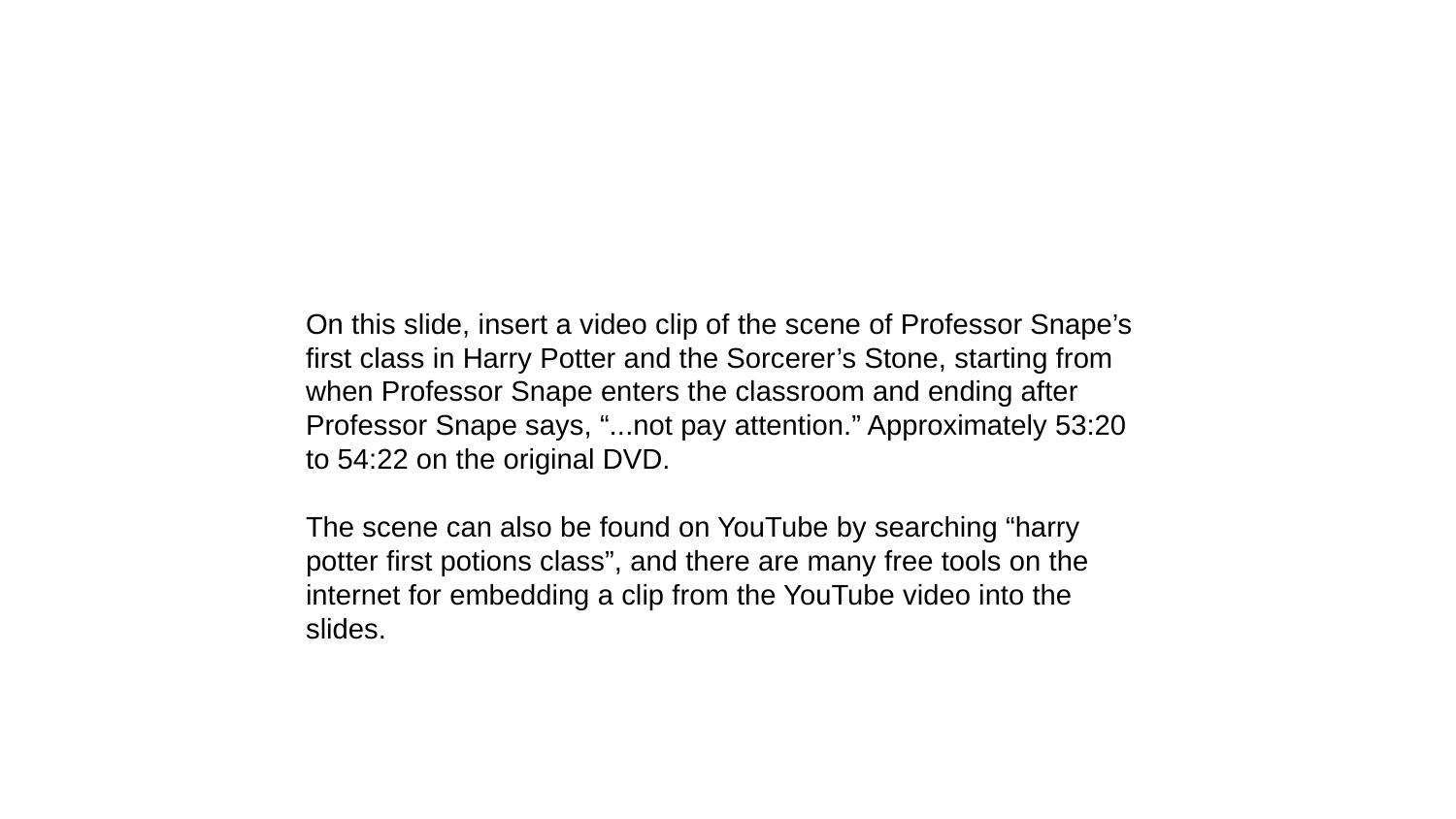

On this slide, insert a video clip of the scene of Professor Snape’s first class in Harry Potter and the Sorcerer’s Stone, starting from when Professor Snape enters the classroom and ending after Professor Snape says, “...not pay attention.” Approximately 53:20 to 54:22 on the original DVD.
The scene can also be found on YouTube by searching “harry potter first potions class”, and there are many free tools on the internet for embedding a clip from the YouTube video into the slides.

## Slide 12
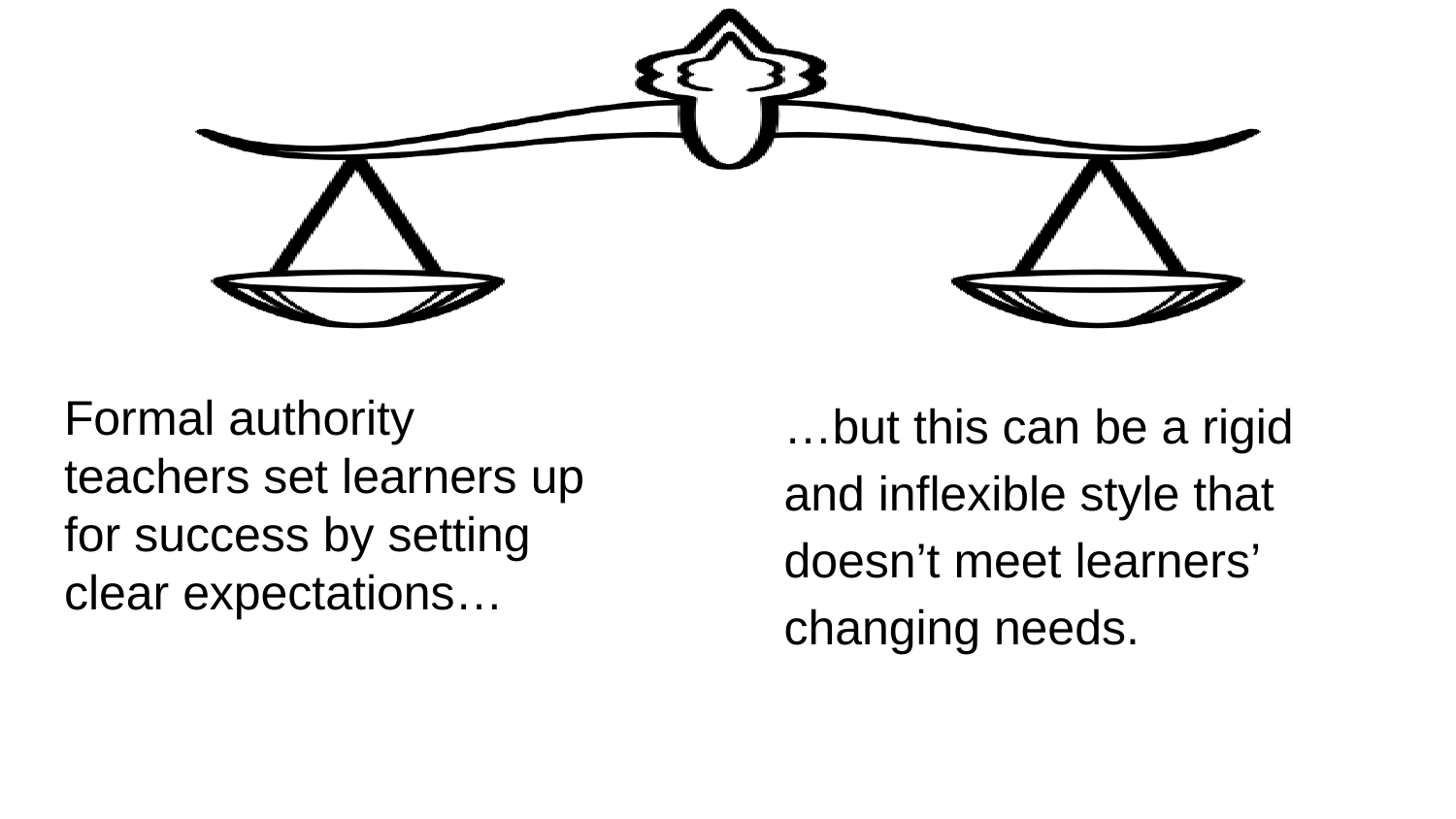

Formal authority teachers set learners up for success by setting clear expectations…
…but this can be a rigid and inflexible style that doesn’t meet learners’ changing needs.

## Slide 13
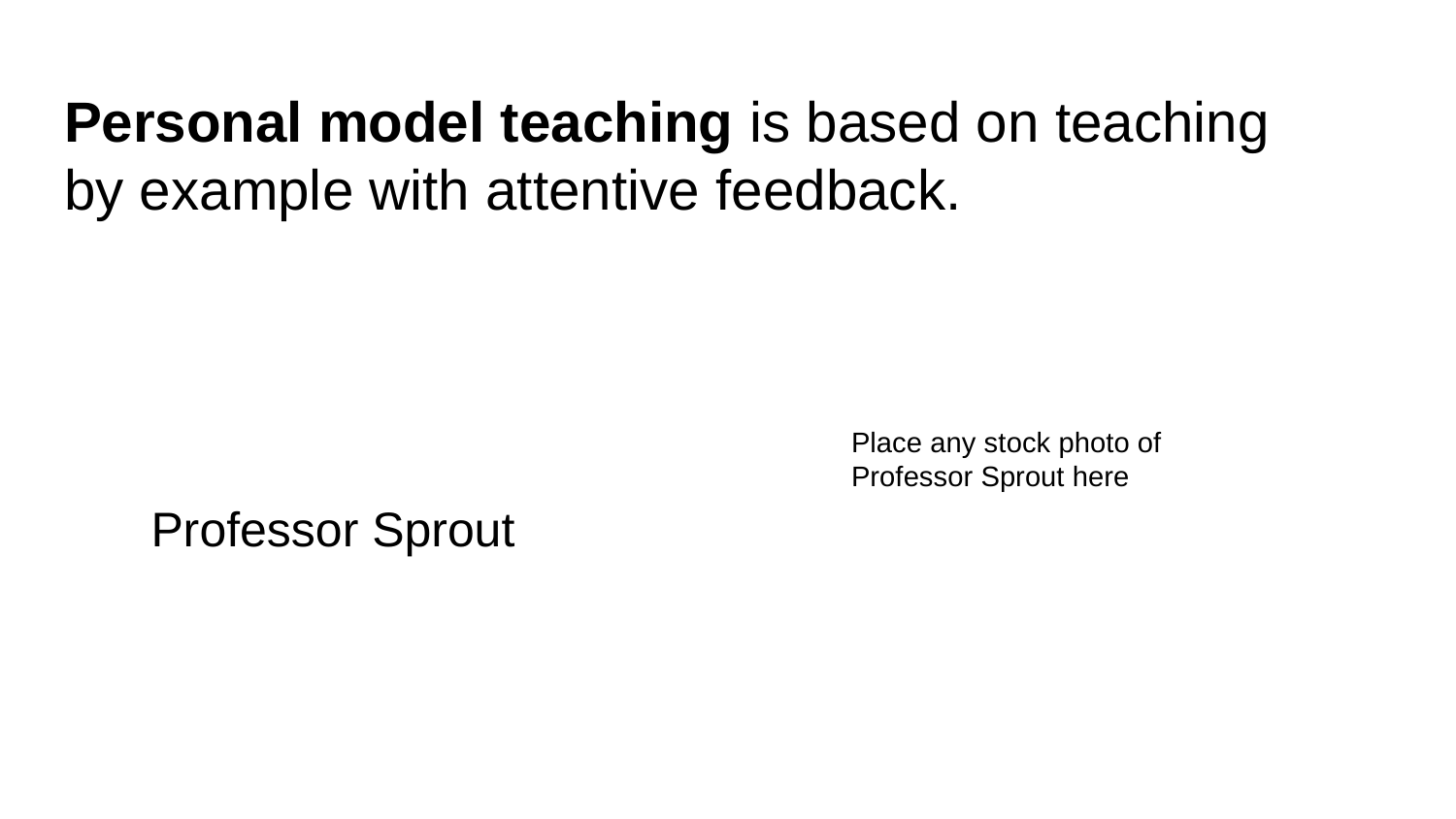

# Personal model teaching is based on teaching by example with attentive feedback.
Place any stock photo of Professor Sprout here
Professor Sprout

## Slide 14
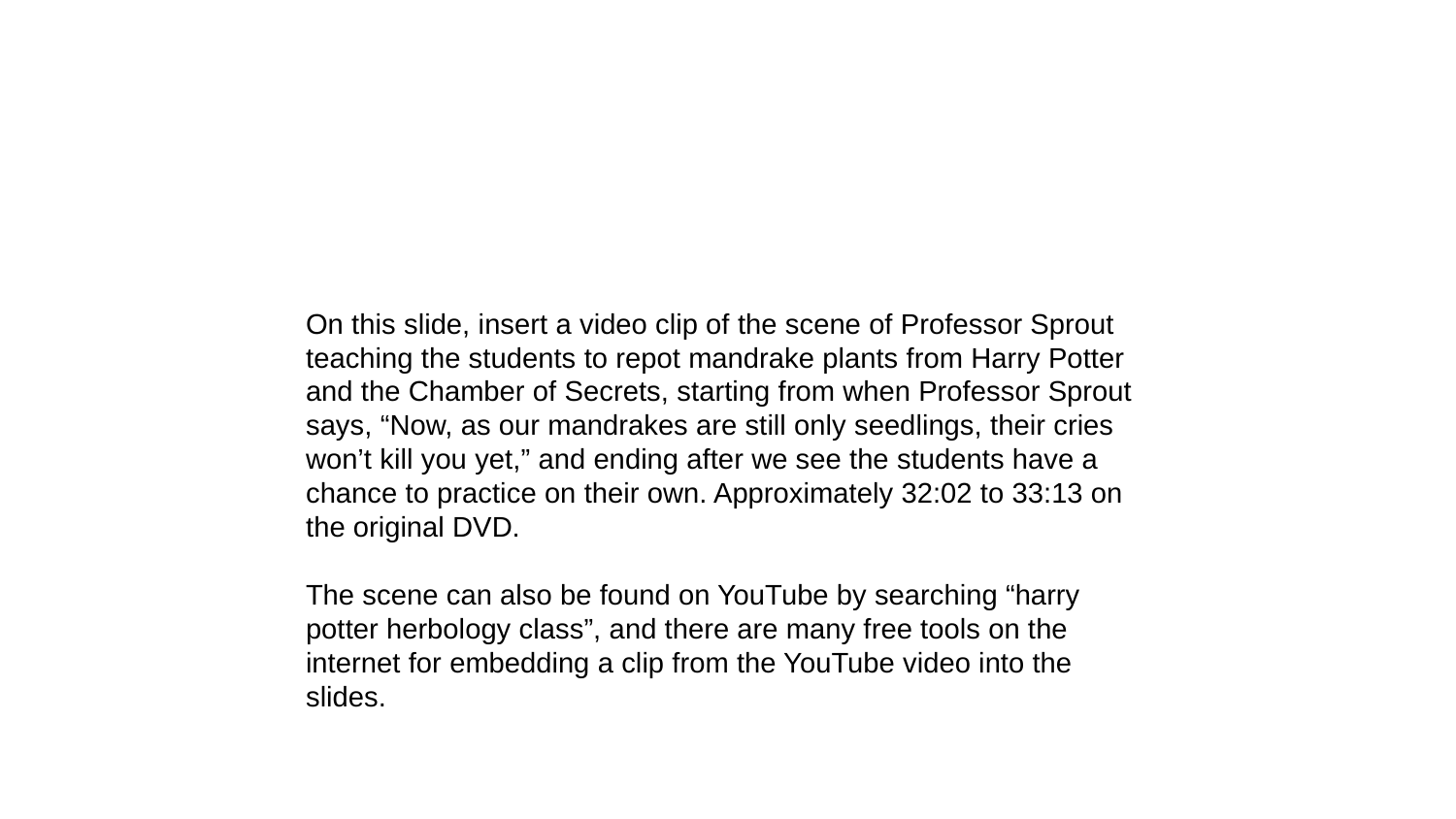

On this slide, insert a video clip of the scene of Professor Sprout teaching the students to repot mandrake plants from Harry Potter and the Chamber of Secrets, starting from when Professor Sprout says, “Now, as our mandrakes are still only seedlings, their cries won’t kill you yet,” and ending after we see the students have a chance to practice on their own. Approximately 32:02 to 33:13 on the original DVD.
The scene can also be found on YouTube by searching “harry potter herbology class”, and there are many free tools on the internet for embedding a clip from the YouTube video into the slides.

## Slide 15
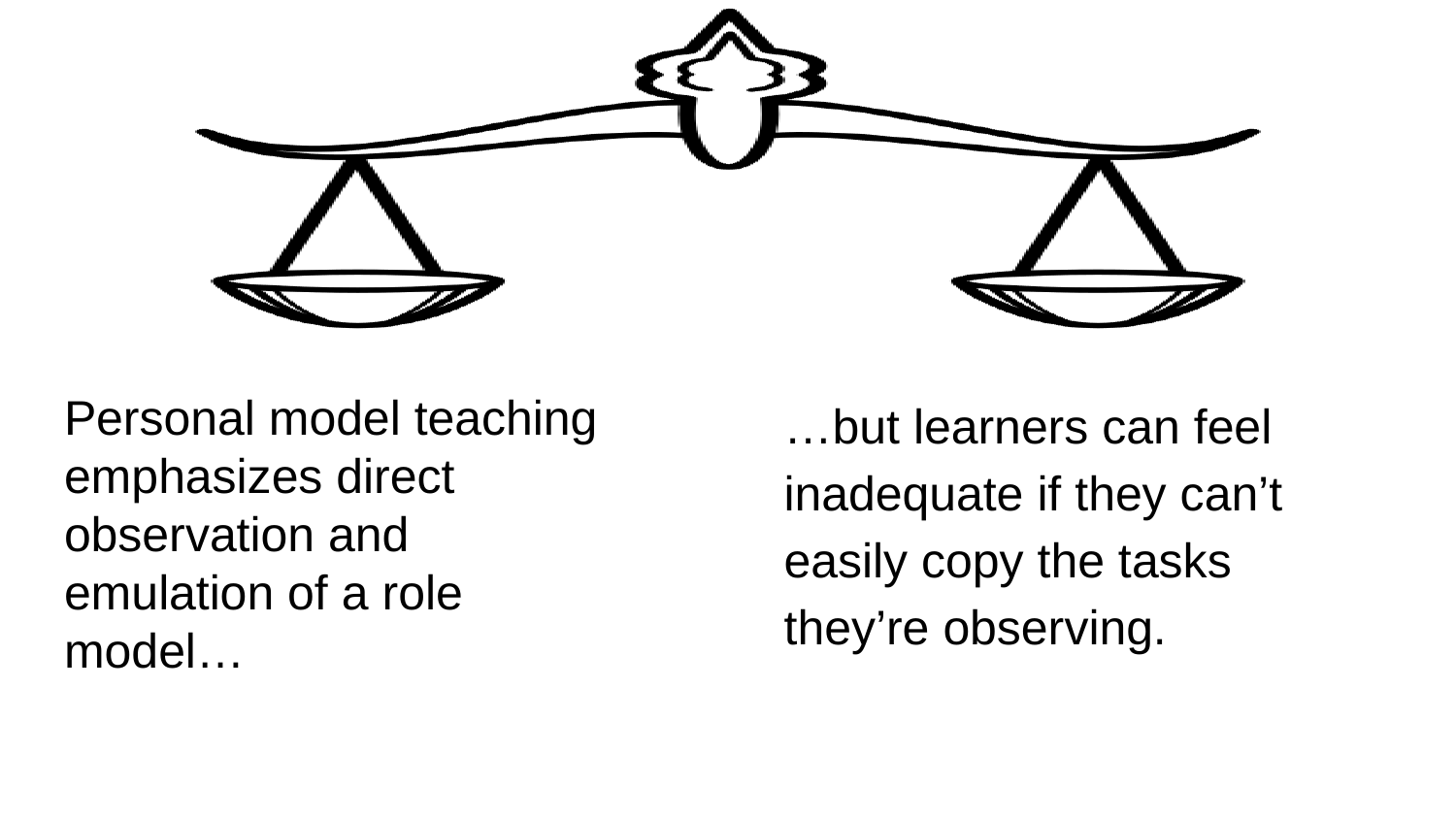

Personal model teaching emphasizes direct observation and emulation of a role model…
…but learners can feel inadequate if they can’t easily copy the tasks they’re observing.

## Slide 16
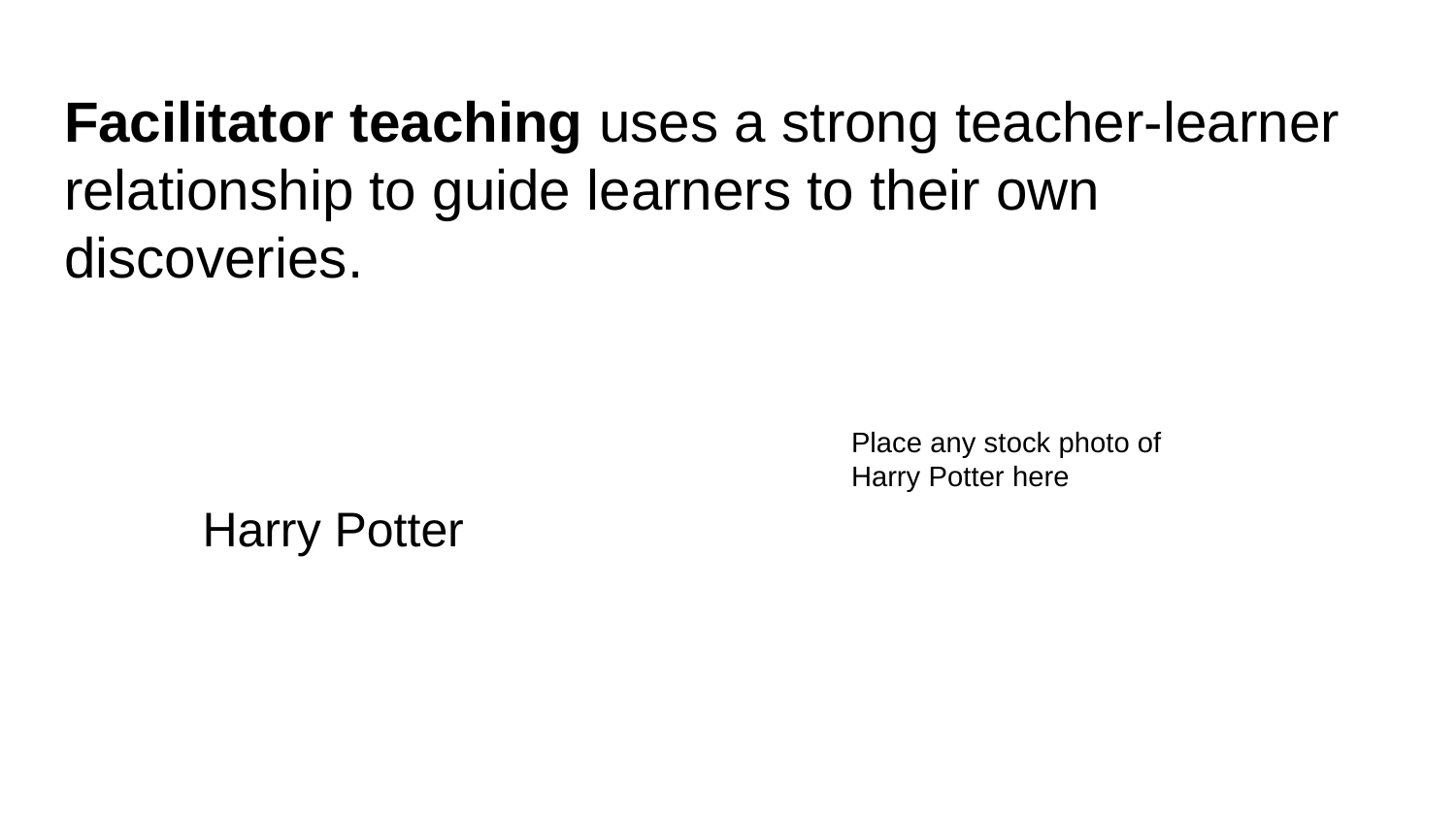

# Facilitator teaching uses a strong teacher-learner relationship to guide learners to their own discoveries.
Place any stock photo of Harry Potter here
Harry Potter

## Slide 17
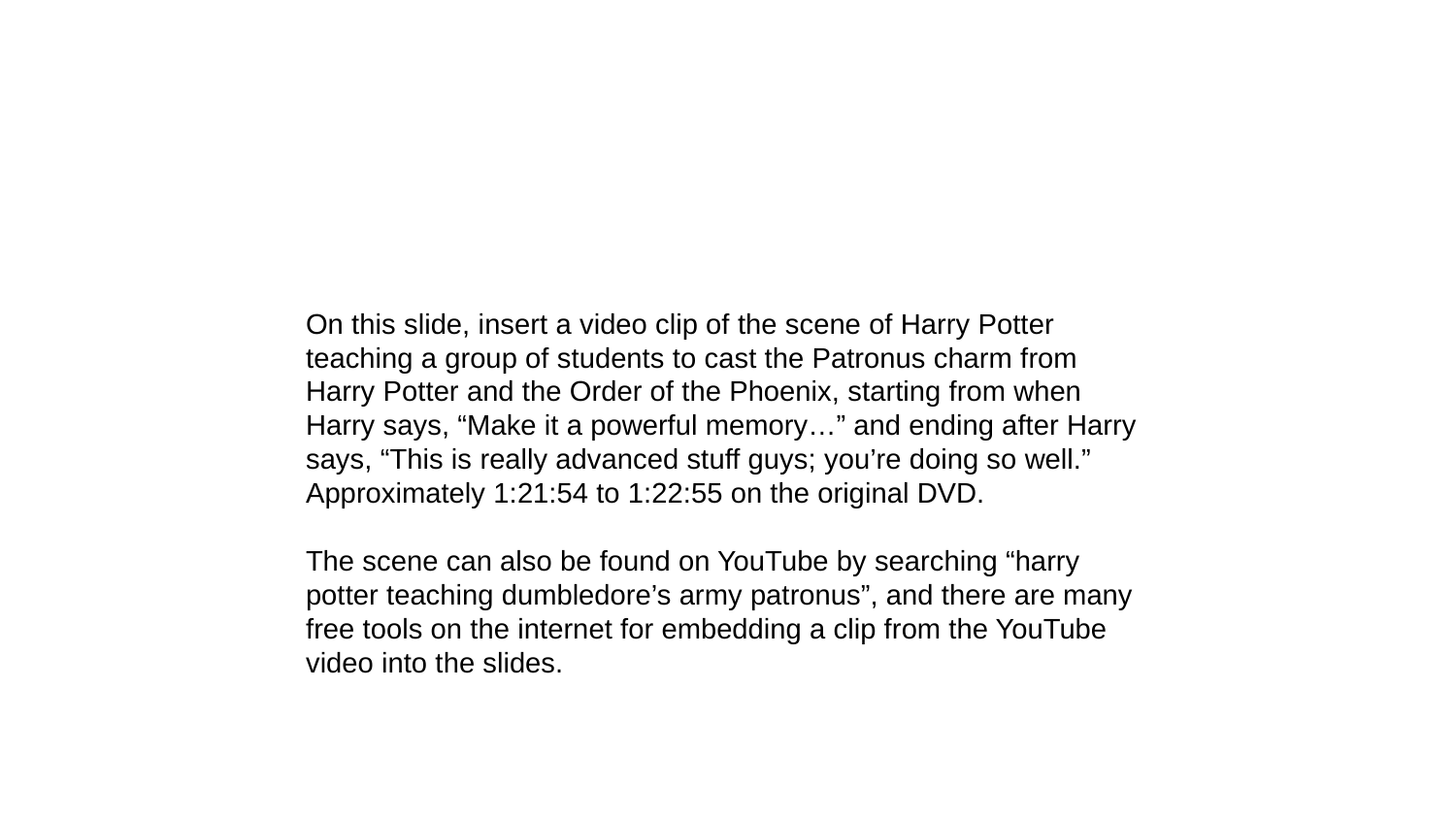

On this slide, insert a video clip of the scene of Harry Potter teaching a group of students to cast the Patronus charm from Harry Potter and the Order of the Phoenix, starting from when Harry says, “Make it a powerful memory…” and ending after Harry says, “This is really advanced stuff guys; you’re doing so well.” Approximately 1:21:54 to 1:22:55 on the original DVD.
The scene can also be found on YouTube by searching “harry potter teaching dumbledore’s army patronus”, and there are many free tools on the internet for embedding a clip from the YouTube video into the slides.

## Slide 18
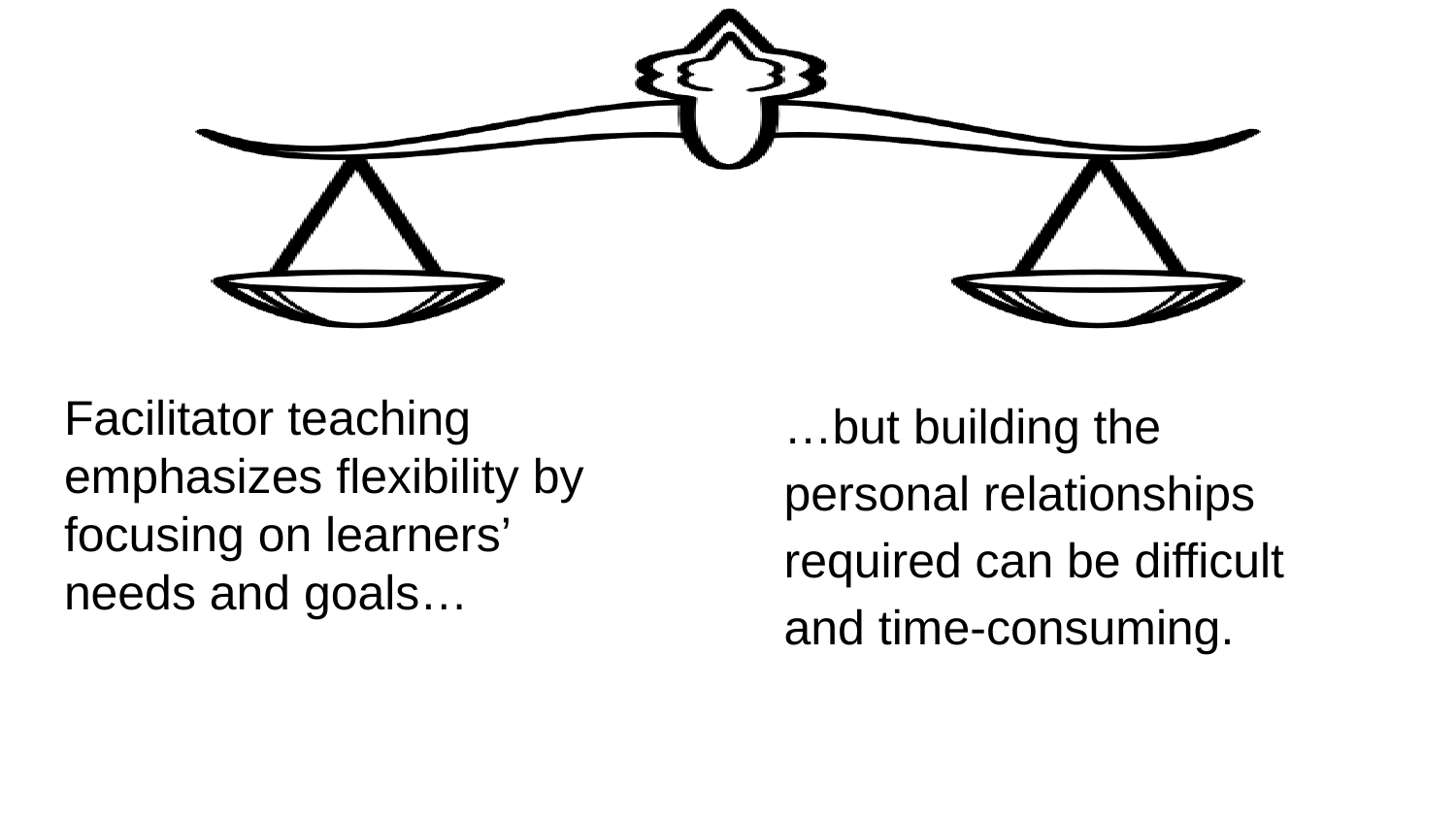

Facilitator teaching emphasizes flexibility by focusing on learners’ needs and goals…
…but building the personal relationships required can be difficult and time-consuming.

## Slide 19
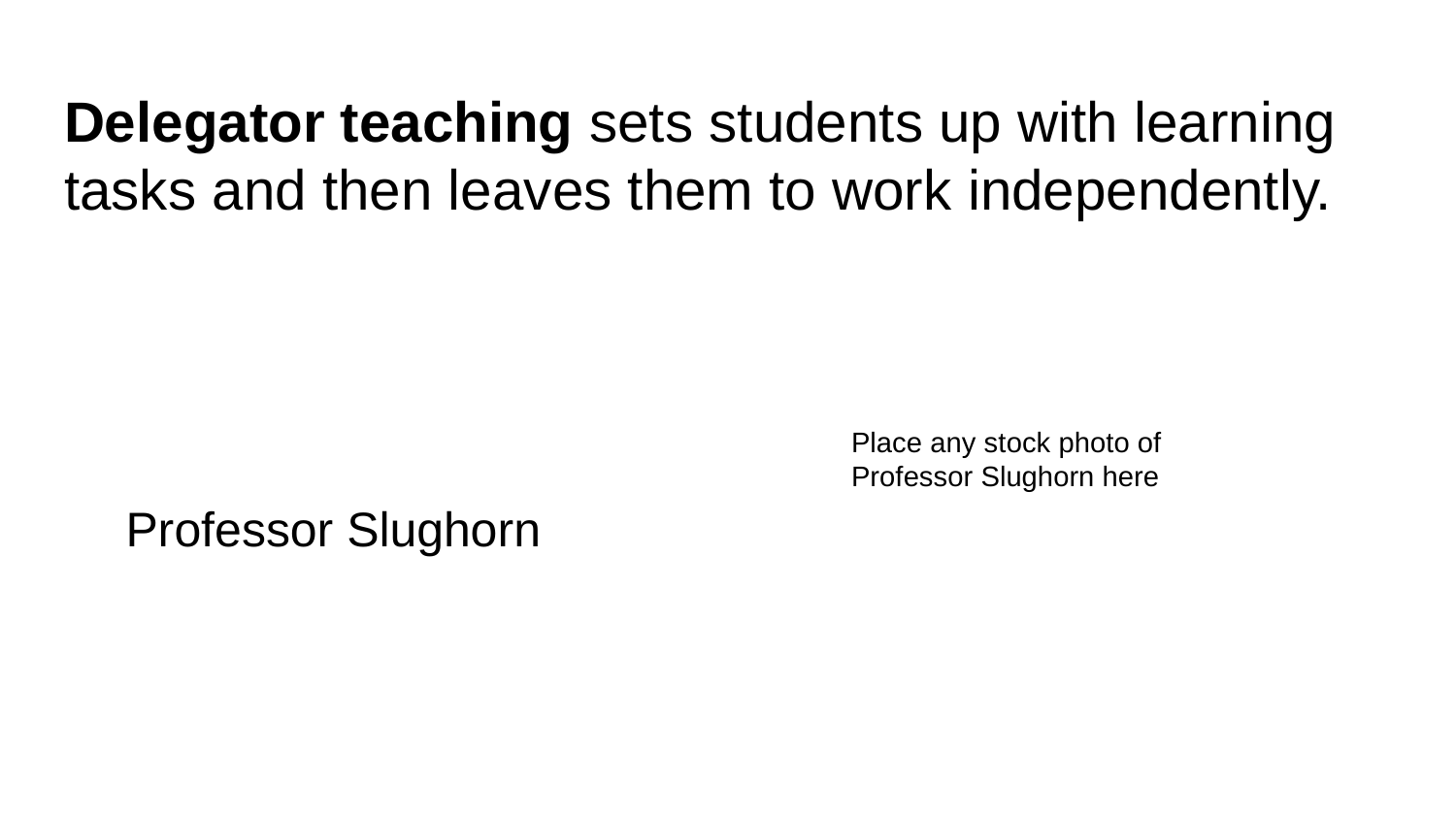

# Delegator teaching sets students up with learning tasks and then leaves them to work independently.
Place any stock photo of Professor Slughorn here
Professor Slughorn

## Slide 20
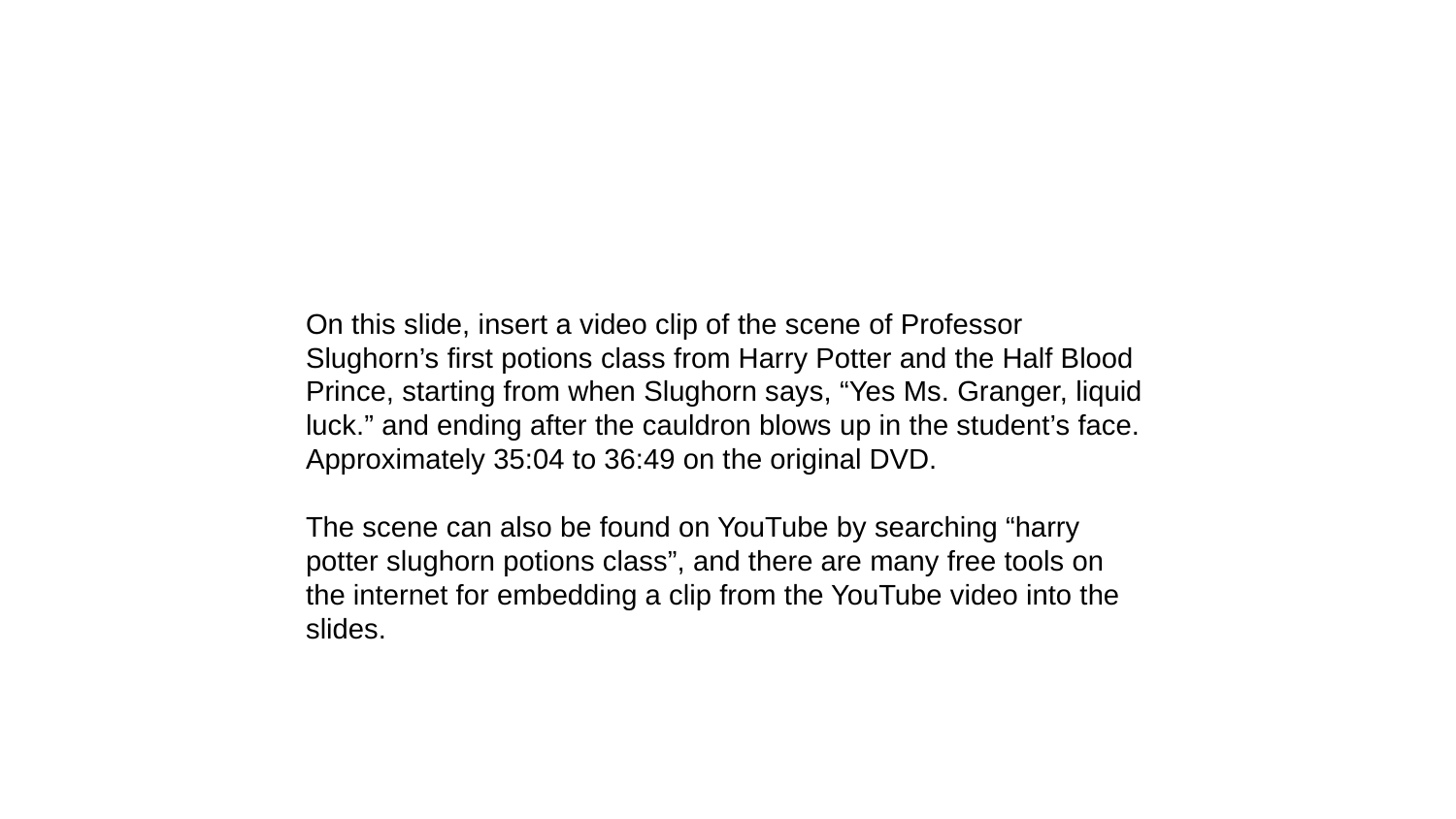

On this slide, insert a video clip of the scene of Professor Slughorn’s first potions class from Harry Potter and the Half Blood Prince, starting from when Slughorn says, “Yes Ms. Granger, liquid luck.” and ending after the cauldron blows up in the student’s face. Approximately 35:04 to 36:49 on the original DVD.
The scene can also be found on YouTube by searching “harry potter slughorn potions class”, and there are many free tools on the internet for embedding a clip from the YouTube video into the slides.

## Slide 21
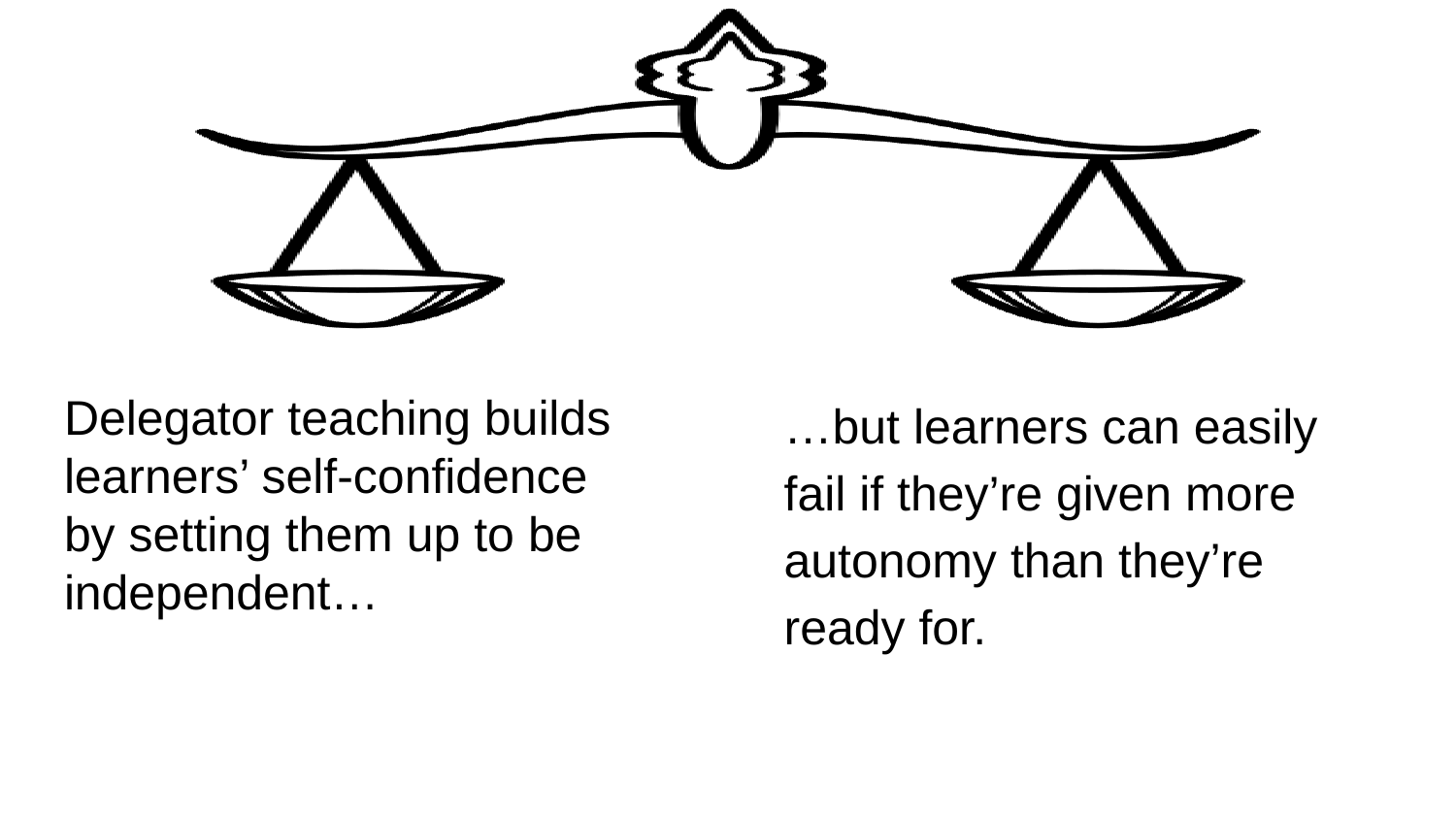

Delegator teaching builds learners’ self-confidence by setting them up to be independent…
…but learners can easily fail if they’re given more autonomy than they’re ready for.

## Slide 22
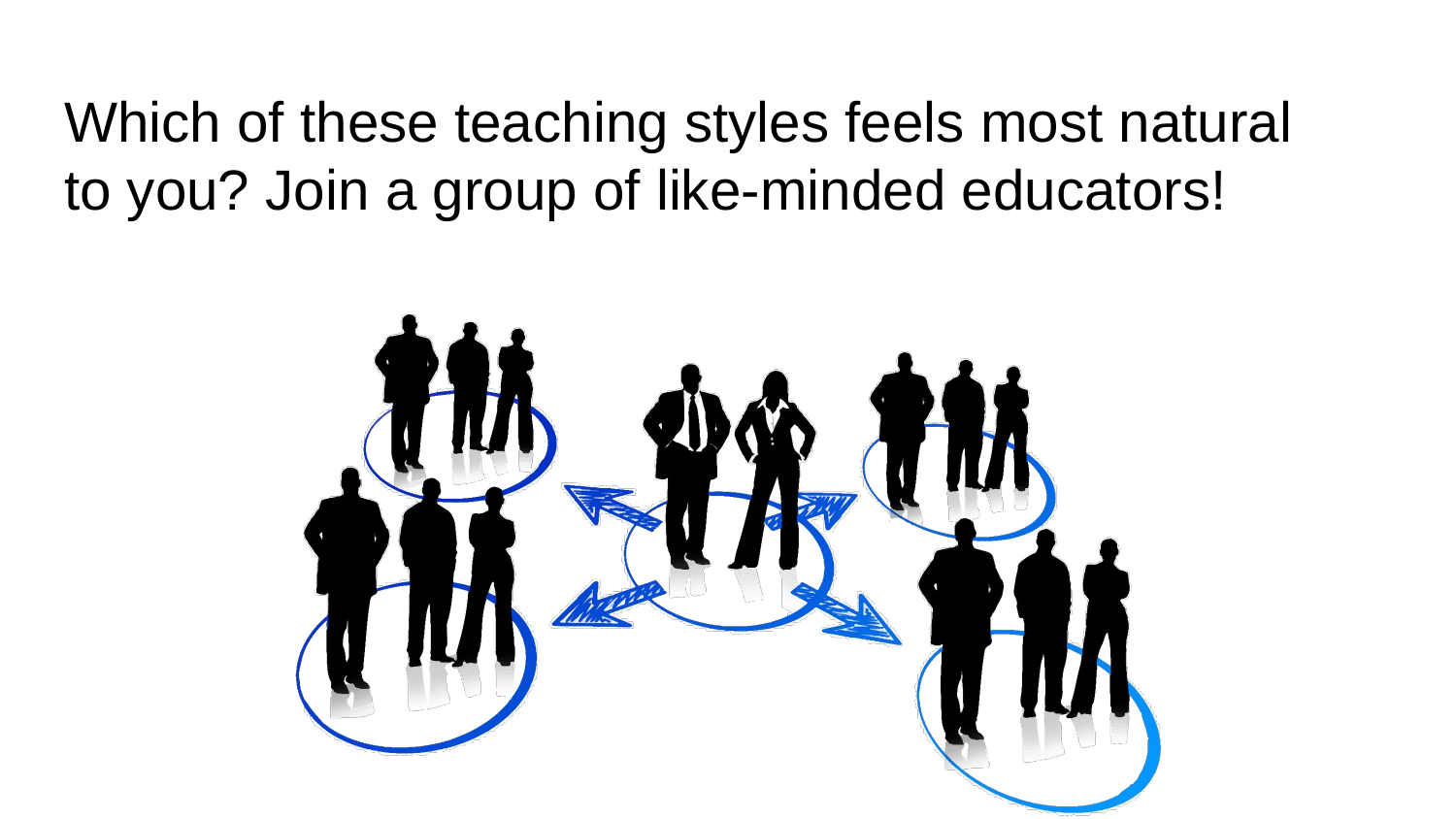

Which of these teaching styles feels most natural
to you? Join a group of like-minded educators!

## Slide 23
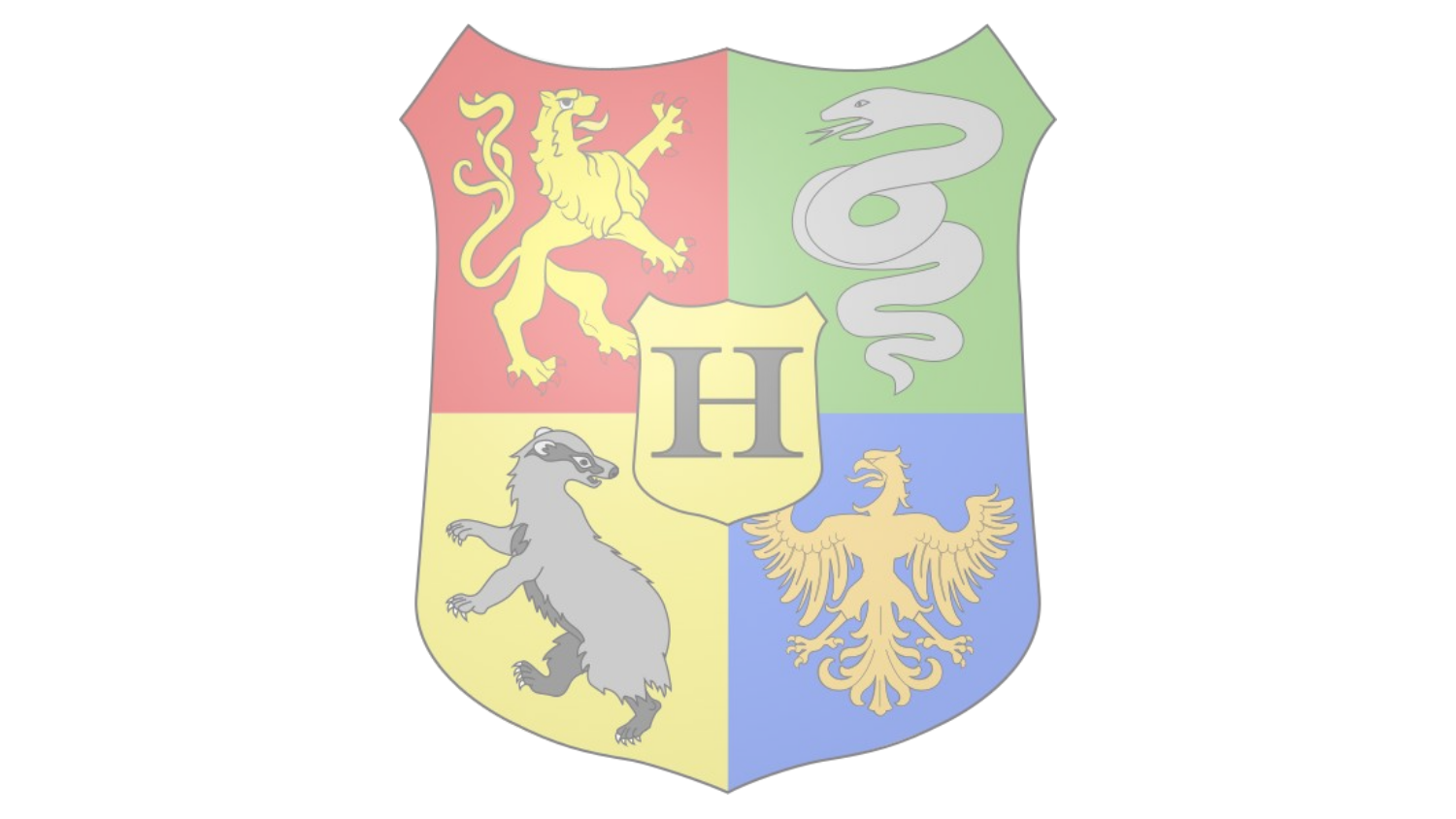

## Slide 24
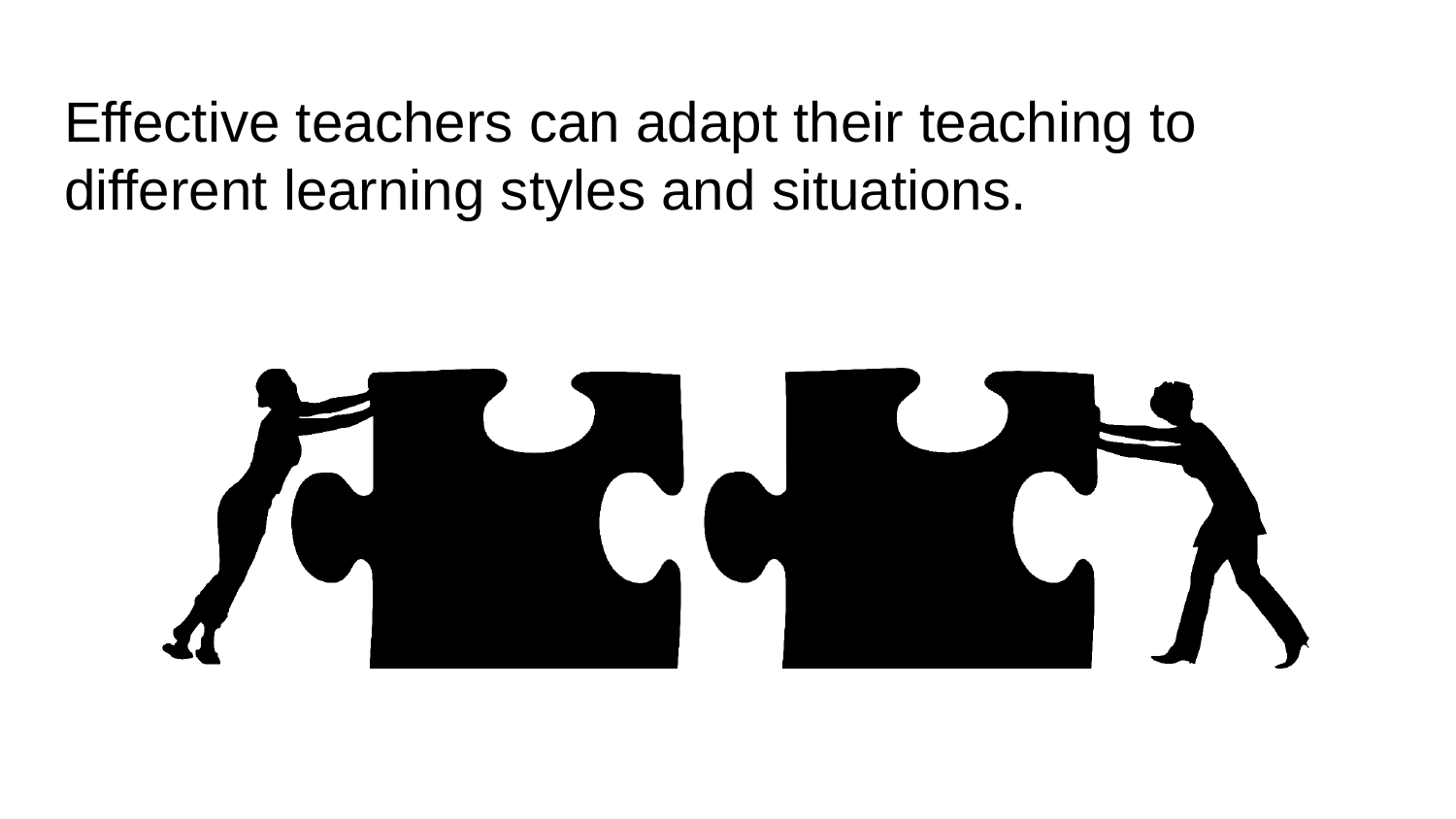

# Effective teachers can adapt their teaching to different learning styles and situations.

## Slide 25
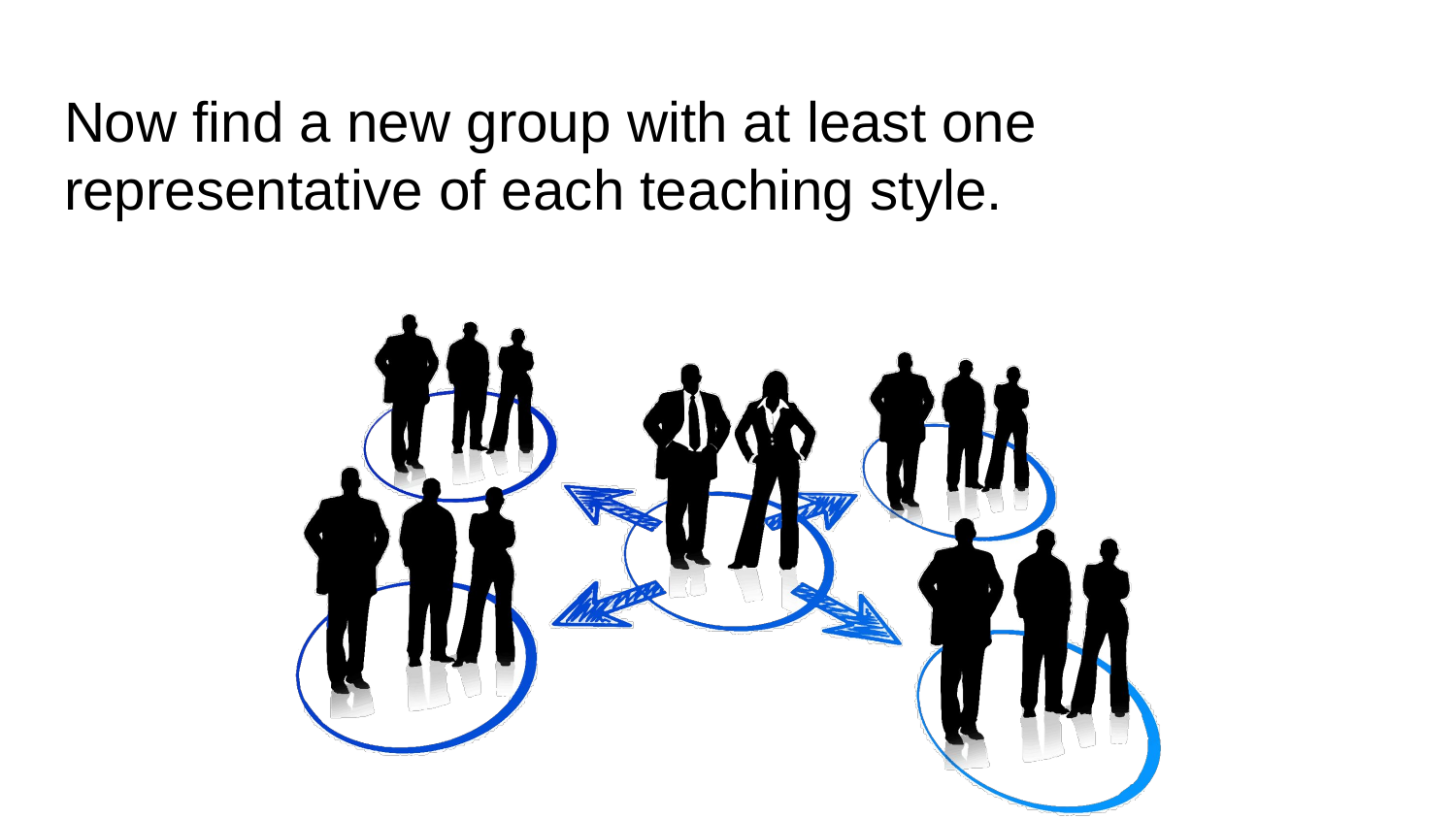

# Now find a new group with at least one representative of each teaching style.

## Slide 26
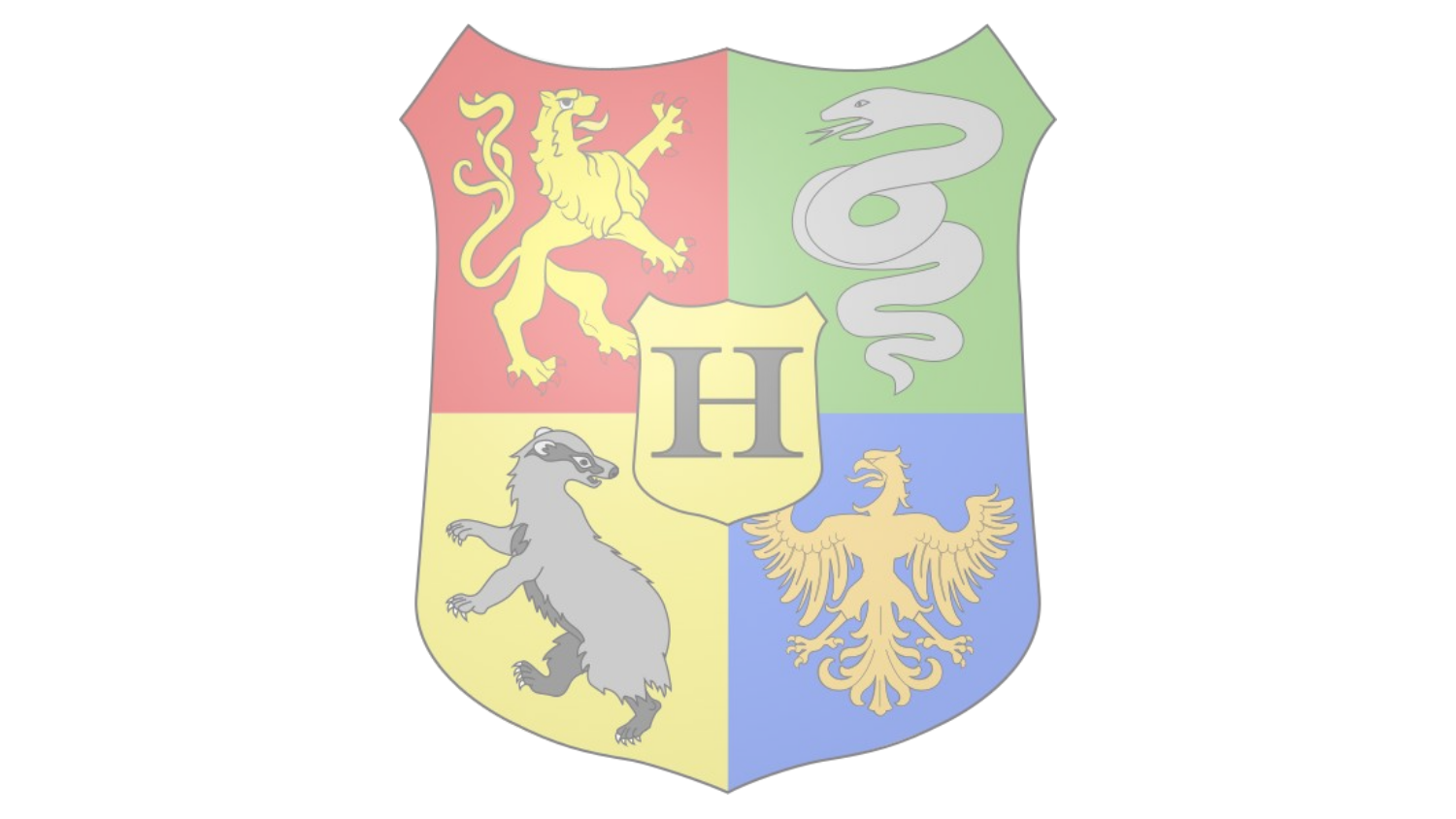

## Slide 27
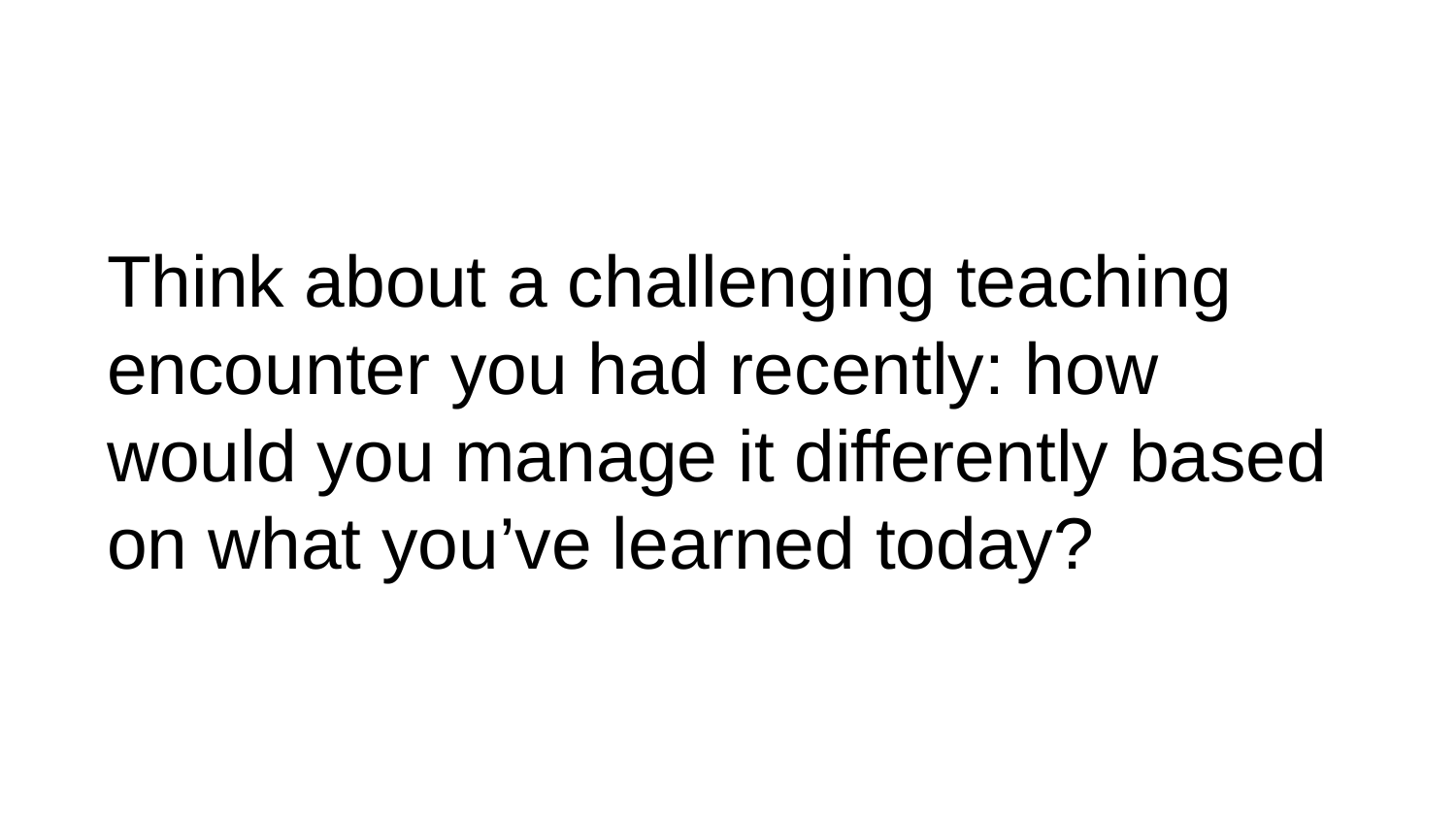

# Think about a challenging teaching encounter you had recently: how would you manage it differently based on what you’ve learned today?

## Slide 28
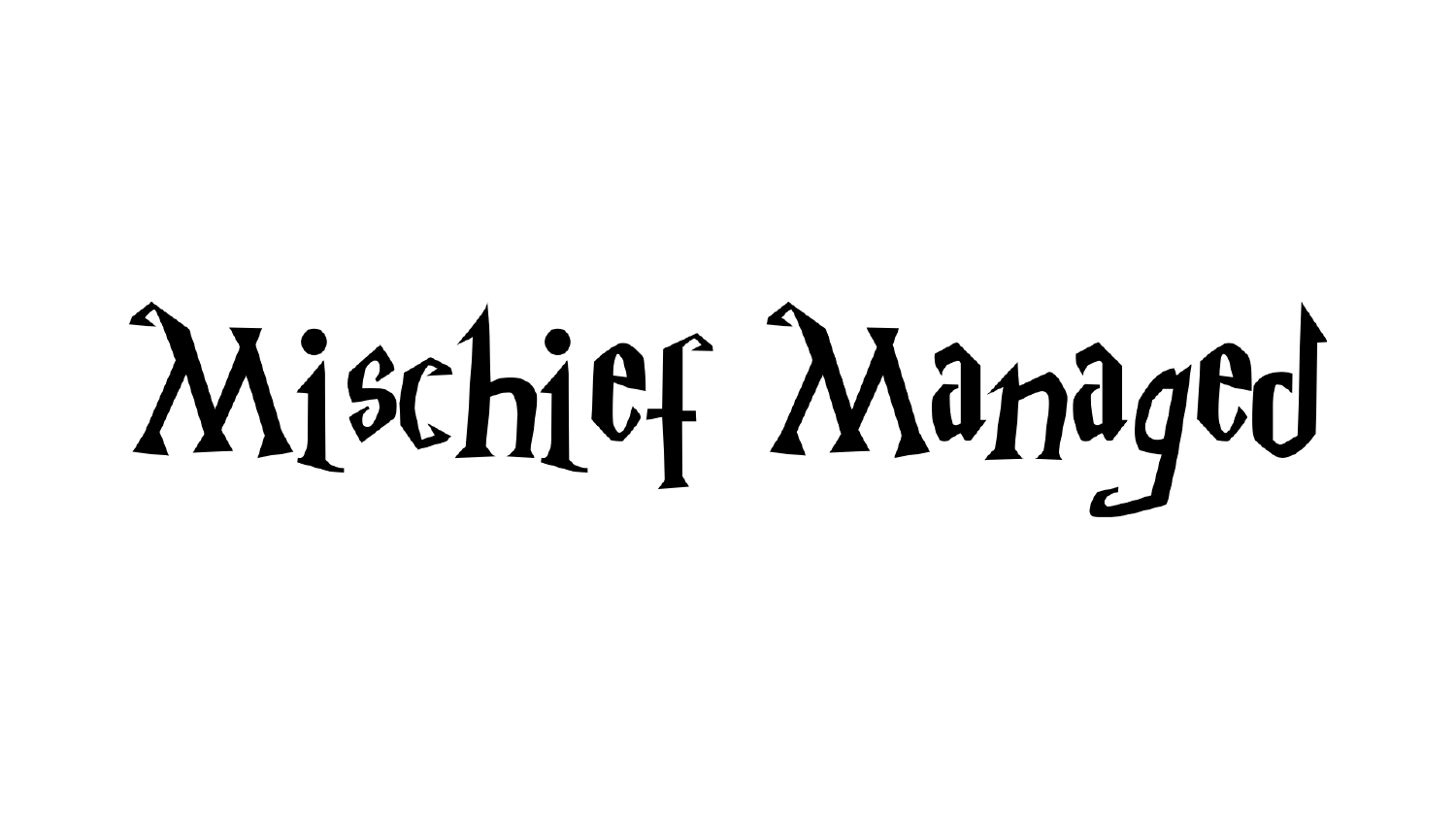

Supplement: Supplementary file 1 — Harry Potter Teaching Styles Handout.docxHarry Potter Teaching Styles Workshop.pptxDiscussion Cases.docxFacilitator Guide.docxWorkshop Evaluation.docx [file mep_2374-8265.11571-s001.zip › B. Harry Potter Teaching Styles Workshop.pptx]
