# Supplementary material for: An Interactive Workshop to Enhance Teaching Skills Through Understanding Teaching Styles
Source: MedEdPORTAL. 2026 Jan 20;22:11571. doi: 10.15766/mep_2374-8265.11571 (PMC12816393; doi:10.15766/mep_2374-8265.11571)
Supplement: Supplementary file 1 — Harry Potter Teaching Styles Handout.docxHarry Potter Teaching Styles Workshop.pptxDiscussion Cases.docxFacilitator Guide.docxWorkshop Evaluation.docx [file mep_2374-8265.11571-s001.zip › C. Discussion Cases.docx]

Lessons from the Hogwarts School of Faculty Development Teaching Case Scenarios 1 & 2

1. You are the hospitalist on service for the week and it has been busy! You have a large team with 1 PGY3, 2 pediatric PGY1s and 1 family medicine intern. There is also a sub-intern and 2 third-year medical students on the team. By the third day of rounding, you have noted that everyone is reporting detailed patient data - but they are not keeping up and thinking ahead. It seems that assessments are consistently incorrect because of the use of outdated information. You get the sense that no one is doing any reading about their patients (possibly because of the high patient volume).

2. It’s February in the newborn nursery and you are working with one of the PGY1s that you have worked with previously. Back in August, you remember this PGY1 being very good. They were able to accomplish their work relatively efficiently. They were very receptive to feedback. Your immediate impression now is that they don’t seem to have made any progress. It’s 6 months later and you don’t know if your expectations have changed - or if they truly haven’t progressed. The nursery is very busy this week and you both have >20 babies to see each day. By the end of rounds, you are limited in the time you have to work with this PGY1.

**Discussion questions to consider**

- Focusing on your group’s natural teaching style, how would you approach this situation?

- What are the strengths of your natural teaching style in this situation?

- What are the weaknesses of your natural teaching style in this situation?

Lessons from the Hogwarts School of Faculty Development Teaching Case Scenarios 3 & 4

3. It is Monday and you know that this will be an extremely busy inpatient service week for you. There is no way that you will be able to round on all of these patients unless you are very efficient. You typically have the PGY-3 on the team lead rounds so that they have the opportunity to teach and gain some leadership experience, but you are worried that having the PGY-3 lead rounds will make rounds go on too long.

4. You are in the busy outpatient clinic and you have had a third-year medical student rotating with you for the past few days. You have noted that this student has been very engaged. They eagerly go evaluate patients and present to you. They are clearly learning from each case as you have seen their pediatric histories and

assessments improve during the past few days. It has been so hectic though that you have not been able to teach at all in the way that you normally like to. You normally like to sit down after a patient encounter and discuss a particular aspect of it with a student because you feel that you can really get a sense of the student’s

understanding of the case this way, but given the heavy patient load, it seems like that will not happen today.

**Discussion questions to consider**

- What is your individual natural teaching style, and how would you approach this situation in that style?

- What are the strengths of your natural teaching style in this situation?

- What are the weaknesses of your natural teaching style in this situation?

- If your preferred teaching style may not work well in this situation, what elements of what other styles would you use?
