# Supplementary material for: An Interactive Workshop to Enhance Teaching Skills Through Understanding Teaching Styles
Source: MedEdPORTAL. 2026 Jan 20;22:11571. doi: 10.15766/mep_2374-8265.11571 (PMC12816393; doi:10.15766/mep_2374-8265.11571)
Supplement: Supplementary file 1 — Harry Potter Teaching Styles Handout.docxHarry Potter Teaching Styles Workshop.pptxDiscussion Cases.docxFacilitator Guide.docxWorkshop Evaluation.docx [file mep_2374-8265.11571-s001.zip › D. Facilitator Guide.docx]

**Facilitator Guide to accompany**

***An Interactive Workshop to Enhance Teaching Skills Through Understanding Teaching Styles, or Lessons from the Hogwarts School of Faculty Development***

**Preparation and Personnel:**

- Workshop slides
- Obtain video clips for each teaching style (see Appendix A).
- Ideally, you will have 5 facilitators, one for each teaching style. However, if you have fewer than 5, the available facilitators can circulate among the groups during the breakout sessions.
- Slides can be divided among facilitators as desired, but all facilitators should be familiar with each of the teaching styles to answer questions in breakout sessions.

**Equipment and Materials:**

- A room that can accommodate participants with tables (or groups of chairs) to allow them to break out into 5 groups
- Computer with Microsoft Office suite and projector
- Harry Potter video clips
- PowerPoint slides: *Lessons from the Hogwarts School of Faculty Development*
- Handout: *Grasha and Reichmann Teaching Styles*
- Facilitator Guide

**Learning Objectives:**

By the end of the workshop, participants will be able to:

1. Identify their natural teaching style based on Grasha and Riechman’s framework
2. Compare and contrast the five teaching styles described by Grasha and Riechman
3. Incorporate elements from each of the five teaching styles to meet particular learner needs or situations

**Framework:**

When preparing and leading this workshop, keep in mind the basic tenet that all five of the Grasha-Riechman teaching styles are equally useful, effective, and appropriate. One style may work better than another in a particular situation, but none of the styles is inherently better than any of the others. In some disciplines, institutions, or cultures, one style may be more socially desirable than others, and it is important to resist that bias, both as a facilitator and for the workshop participants. Remember that social desirability of a particular teaching style may not accurately reflect efficacy.

**Scripts:**

To streamline your preparation and teaching, we have provided a suggested script for the slides in the speaker notes within the slide deck. Additional teaching points for cases in the breakout sessions follow.

**Timeline:**

This workshop was originally designed for 75, and 90-minute timeframes. We later developed a shortened version to fit in 60-minutes. The 90-minute agenda could also be easily expanded to 120 minutes by allowing more time for discussion.

**60-minute agenda**

| Time | Activity / Content | Slides |
| --- | --- | --- |
| 0:00-0:10 | Introduction of Workshop and Grasha Reichman Teaching Styles | 1-6 |
| 0:10-0:14 | Expert Teaching Style | 7-9 |
| 0:14-0:18 | Formal Authority Teaching Style | 10-12 |
| 0:18-0:22 | Personal Model Teaching Style | 13-15 |
| 0:22-0:26 | Facilitator Teaching Style | 16-18 |
| 0:26-0:30 | Delegator Teaching Style | 19-21 |
|  | *Skip slides 22-23 and Breakout Session I Use all four cases for Breakout Session II* |  |
| 0:30-0:50 | Breakout Session | 24-26 |
| 0:50-0:60 | Wrap up | 27-28 |

**75-minute agenda**

| Time | Activity / Content | Slides |
| --- | --- | --- |
| 0:00-0:10 | Introduction of Workshop and Grasha Reichman Teaching Styles | 1-6 |
| 0:10-0:14 | Expert Teaching Style | 7-9 |
| 0:14-0:18 | Formal Authority Teaching Style | 10-12 |
| 0:18-0:22 | Personal Model Teaching Style | 13-15 |
| 0:22-0:26 | Facilitator Teaching Style | 16-18 |
| 0:26-0:30 | Delegator Teaching Style | 19-21 |
| 0:30-0:45 | Breakout Session I | 22-23 |
| 0:45-0:47 | Adapting Teaching Styles |  |
| 0:47-0:65 | Breakout Session II | 24-26 |
| 0:65-0:75 | Wrap up | 27-28 |

**90-minute agenda**

| Time | Activity / Content | Slides |
| --- | --- | --- |
| 0:00-0:10 | Introduction of Workshop and Grasha Reichman Teaching Styles | 1-6 |
| 0:10-0:15 | Expert Teaching Style | 7-9 |
| 0:15-0:20 | Formal Authority Teaching Style | 10-12 |
| 0:20-0:25 | Personal Model Teaching Style | 13-15 |
| 0:25-0:30 | Facilitator Teaching Style | 16-18 |
| 0:30-0:35 | Delegator Teaching Style | 19-21 |
| 0:35-0:50 | Breakout Session I | 22-23 |
| 0:50-0:55 | Adapting Teaching Styles |  |
| 0:55-0:80 | Breakout Session II | 24-26 |
| 0:80-0:90 | Wrap up | 27-28 |

**Case Discussion Points**

These cases and discussion questions appear in Appendix B. Here, we have included suggestions on how to adapt the cases to different specialties or contexts and possible responses reflecting how someone using each of the five styles might approach each case.

***Case 1***

*You are the hospitalist on service for the week and it has been busy! You have a large team with 1 PGY3, 2 pediatric PGY1s and 1 family medicine intern. There is also a sub-intern and 2 third-year medical students on the team. By the third day of rounding, you have noted that everyone is reporting detailed patient data - but they are not keeping up and thinking ahead. It seems that assessments are consistently incorrect because of the use of outdated information. You get the sense that no one is doing any reading about their patients (possibly because of the high patient volume).*

**Tips for adjusting this case:**
You may adjust the specialty for each resident or the level of training. The main point is to have some residents on the team who are more experienced and familiar with the specialty and then a very junior trainee who has no experience in that specialty. You can consider using interns from preliminary programs or transitional year programs, or medical students for this teaching scenario.

| Teaching Style | Sample Responses |
| --- | --- |
| Expert | **Approach**: Verbalize/teach team members about the correct assessments for each patient.  **Strengths**: Learners are explicitly taught the right answer for each patient.  **Weaknesses**: Limited insight as to why the learners are struggling to gather information and why they are not thinking ahead about the clinical picture. It is also easy to just provide the correct “answers” and not explain the rationale behind each assessment/plan. |
| Formal Authority | **Approach**: Set expectations that learners need to collect xyz information and use the most recent information to generate appropriate and up-to-date assessments.  **Strengths**: Provides explicit guidance and expectations to the learners on how to provide patient care.  **Weaknesses**: This style does not explore other reasons as to why the learners are not keeping up with their patients, nor does it help learners overcome those barriers. |
| Personal Model | **Approach**: Demonstrate how you would go about collecting data and generating relevant and accurate assessments. Afterwards, have the learners try it and show you.  **Strengths**: You are demonstrating how to complete the tasks consistent with your preferred approach.  **Weaknesses**: Your method may not work well for everyone as each person may approach or think about things differently. |
| Facilitator | **Approach**: Ask learners to reflect on how they’re doing and what they may be struggling with. Guide the learner to create a plan that will help them obtain the correct information and assessments.  **Strengths**: Promotes self-reflection and self-directed learning by the learner.  **Weaknesses**: This can be more difficult if learners have limited insight. This also requires learners to trust that the teacher will not judge if learners admit to “flaws” or “areas to work on.” Time is required to build that trust and relationship. |
| Delegator | **Approach**: Continue to tell the learners to gather information and form assessments.  **Strengths**: Provides autonomy and allows a chance for learners to do things “their way.”  **Weaknesses**: As illustrated in this case, the learners are not able to do these tasks correctly on their own. Leaving them to do it without further instruction will continue to perpetuate this cycle. |

***Case 2***

*It’s February in the newborn nursery and you are working with one of the PGY1s that you have worked with previously. Back in August, you remember this PGY1 being very good. They were able to accomplish their work relatively efficiently. They were very receptive to feedback. Your immediate impression now is that they don’t seem to have made any progress. It’s 6 months later and you don’t know if your expectations have changed - or if they truly haven’t progressed. The nursery is very busy this week and you both have >20 babies to see each day. By the end of rounds, you are limited in the time you have to work with this PGY1.*

**Tips for adjusting this case:** Change the setting to any busy rotation where a resident may rotate through multiple times or work with the same faculty multiple times.

| Teaching Style | Sample Responses |
| --- | --- |
| Expert | **Approach**: Teach the PGY1 how to round on newborns efficiently.  **Strengths**: PGY1 may have forgotten how to round on newborns, so doing this provides a refresher.  **Weaknesses**: You are assuming that their knowledge base is incorrect, but the inefficiency/lack of progression could actually be related to something else. |
| Formal Authority | **Approach**: Set expectations that tasks must be completed within a certain time period and provide guidance for the expected time for each component.  **Strengths**: Allows for the PGY-1 to recognize that there is a time limit for certain tasks and learn to prioritize tasks.  **Weaknesses**: Knowing how long something should take does not automatically mean that the tasks can be completed if, for instance, the PGY-1 does not know how to do it. |
| Personal Model | **Approach**: Demonstrate how you perform newborn rounds and then have the PGY-1 demonstrate it for you.  **Strengths**: You model the ideal way to proceed with newborn rounds and then ask the PGY-1 to practice the approach. You can give specific feedback on areas where the PGY-1 needs help.  **Weaknesses**: When this is such a busy service, you may not be able to fully demonstrate, observe, and provide feedback. |
| Facilitator | **Approach**: Ask PGY-1 to reflect on how newborn rounds are going and where they’re struggling with. Guide the learner to create a plan that they feel will help them be more efficient.  **Strengths**: Promotes self-reflection and self-directed learning by the learner.  **Weaknesses**: This requires time to rebuild trust and for the learner to reflect and self-assess. The learner may be unable to do so, especially on a busy service. |
| Delegator | **Approach**: Continue to tell the learner to round on newborns.  **Strengths**: Provides autonomy and allows a chance for learners to do things “their way.”  **Weaknesses**: As is evident in this case, the learner is inefficient on their own. Leaving them to do it without further instruction will continue to perpetuate this cycle. |

***Case 3***

*It is Monday and you know that this will be an extremely busy inpatient service week for you. There is no way that you will be able to round on all of these patients unless you are very efficient. You typically have the PGY-3 on the team lead rounds so that they have the opportunity to teach and gain some leadership experience, but you are worried that having the PGY-3 lead rounds will make rounds go on too long.*

**Tips for adjusting this case:** The key to this case is balancing efficiency when your patient volume is high against autonomy for the learner(s). This case could be modified to be about any case where a learner might typically take a leadership role, but doing that might be challenging for efficient patient care.

| Teaching Style | Sample Responses |
| --- | --- |
| Expert | **Approach**: Teach the PGY3 how to lead rounds correctly.  **Strengths**: Gives the PGY3 the opportunity to lead the rounds correctly.  **Weaknesses**: It is possible that the PGY3 already knows how to lead rounds, but needs to work on something else. |
| Formal Authority | **Approach**: Set expectations that rounds must occur in a certain time.  **Strengths**: Allows the PGY3 to gain the experience they want and also ensures that the rounds don’t take too long.  **Weaknesses**: Knowing how long something should take does not automatically mean that the PGY3 knows how to do this. |
| Personal Model | **Approach**: Demonstrate for the first patient what efficient rounds look like, then have the PGY3 lead rounds for the next patient, and you provide feedback afterwards.  **Strengths**: You model the ideal way to lead rounds and then ask the PGY-3 to demonstrate it as well. You can give specific feedback on areas where the PGY-3 needs help.  **Weaknesses**: When this is such a busy service, you may not be able to fully demonstrate, observe, and provide feedback. |
| Facilitator | **Approach**: Ask PGY-3 to reflect on what they want to work on for leading rounds. Guide the PGY-3 to create a plan that they feel will help them be more efficient.  **Strengths**: Promotes self-reflection and autonomy in the PGY-3  **Weaknesses**: This requires time for the PGY-3 reflect and trial while you guide, which may not be plausible on a busy service. |
| Delegator | **Approach**: Tell the PGY-3 to lead rounds for half of the patients while you lead rounds for the other half.  **Strengths**: Provides autonomy and allows the PGY3 to lead, while you ensure efficiency occurs by leading the other half of rounds.  **Weaknesses**: PGY3 may not be ready to lead the rounds efficiently. Telling them to do so without providing further instruction may be more detrimental. |

***Case 3***

*You are in the busy outpatient clinic and you have had a third-year medical student rotating with you for the past few days. You have noted that this student has been very engaged. They eagerly go evaluate patients and present to you. They are clearly learning from each case as you have seen their pediatric histories and assessments improve during the past few days. It has been so hectic though that you have not been able to teach at all in the way that you normally like to. You normally like to sit down after a patient encounter and discuss a particular aspect of it with a student because you feel that you can really get a sense of the student’s understanding of the case this way, but given the heavy patient load, it seems like that will not happen today.*

**Tips for adjusting this case:** The key to this case is balancing time for direct teaching against time for efficient patient care. This case could be modified to reflect any scenario that puts those values in tension, e.g. a busy day in the ED or the OR and limited time to sit down and teach.

| Teaching Style | Sample Responses |
| --- | --- |
| Expert | **Approach**: Teach clinical pearls for each patient.  **Strengths**: The student is learning medicine.  **Weaknesses**: It is possible that the student already knows what you are telling them or that they do not have a strong fund of knowledge and doesn’t understand the rationale for what you are teaching them. |
| Formal Authority | **Approach**: Set expectations that you will sometimes have to give delayed feedback when clinic is busy.  **Strengths**: Allows you and the student to be mindful that feedback will come at a given point in time, even if it is delayed.  **Weaknesses**: Feedback is best given in the moment. |
| Personal Model | **Approach**: Instead of giving feedback to the student, you might model the items that you want the student to keep, stop, and start.  **Strengths**: You demonstrate how to implement feedback in real time.  **Weaknesses**: Requires foresight and planning, and may not work as well retroactively. |
| Facilitator | **Approach**: Ask the student what they want to improve and work on.  **Strengths**: Promotes self-reflection in the student and clarifies what guidance is needed in a busy setting.  **Weaknesses**: Requires time to guide the student. |
| Delegator | **Approach**: Tell the student to read up on certain things to improve their knowledge base or watch videos to improve their patient interactions.  **Strengths**: Provides learning goals for the student.  **Weaknesses**: The student may not be able to understand the whys and the correct ways to do things. |
