# Supplementary material for: An Interactive Workshop to Enhance Teaching Skills Through Understanding Teaching Styles
Source: MedEdPORTAL. 2026 Jan 20;22:11571. doi: 10.15766/mep_2374-8265.11571 (PMC12816393; doi:10.15766/mep_2374-8265.11571)
Supplement: Supplementary file 1 — Harry Potter Teaching Styles Handout.docxHarry Potter Teaching Styles Workshop.pptxDiscussion Cases.docxFacilitator Guide.docxWorkshop Evaluation.docx [file mep_2374-8265.11571-s001.zip › E. Workshop Evaluation.docx]

1. After participating in this workshop, how well do you understand your own natural teaching style?
   1. Much better than before
   2. Slightly better than before
   3. Slightly worse than before
   4. Much worse than before
2. Will you change how you teach after participating in this workshop?
   1. Definitely yes
   2. Probably yes
   3. Probably not
   4. Definitely not
3. What is one way that you will change how you teach based on this workshop?
4. What is one thing you've learned from this workshop?
5. How would you recommend we improve this workshop in the future?
6. Any other comments?
